# Deciphering the microbiota-immune axis in adult-onset Still’s disease: evidence from Mendelian randomization and experimental evaluation

**DOI:** 10.3389/fgene.2026.1841584

**Published:** 2026-10-19

**Authors:** Chenlu Li, Fan Yang, Binfeng Wang

**Affiliations:** 1 Department of Gastroenterology, Affiliated Yueqing Hospital, Wenzhou Medical University, Wenzhou, China; 2 Department of Geriatrics, The Second Affiliated Hospital, School of Medicine, Zhejiang University, Hangzhou, Zhejiang, China

**Keywords:** adult-onset Still’s disease, collagen-induced arthritis models, gut microbiota, immune-cell mediator, mediation analysis, Mendelian randomization

## Abstract

**Objectives:**

Adult-onset Still’s disease (AOSD) is a systemic autoinflammatory disorder with unclear etiology. Although gut microbiota may influence immune-mediated diseases, their genetically predicted associations with AOSD remain unclear. This study aims to investigate the potential associations between gut microbiota and AOSD susceptibility and to elucidate the potential mediating roles of immune-cell traits.

**Methods:**

A two-sample Mendelian randomization (MR) framework was employed using genome-wide association study (GWAS) summary statistics from the Dutch Microbiome Project (7,738 individuals), FinnGen cohort (113 AOSD cases and 341,455 controls), and 731 immune-cell traits (3,757 individuals). Potential associations with AOSD susceptibility were assessed using inverse-variance weighted (IVW) and Bayesian weighted MR (BWMR) methods, with Benjamini-Hochberg (BH) false discovery rate (FDR) correction, and were complemented by comprehensive sensitivity analyses to assess robustness. A two-step MR approach was subsequently applied to investigate potential immune-mediated effects. The inflammatory potential of the identified microbial taxa was further examined in collagen-induced arthritis (CIA) mice and microbiota-depleted models treated with *Parabacteroides goldsteinii* (*P. goldsteinii*) and *Alistipes shahii* (*A. shahii*).

**Results:**

MR analysis identified 10 microbial taxa and three metabolic pathways significantly associated with AOSD susceptibility. Notably, the taxa Streptococcaceae, *Streptococcus*, *P. goldsteinii*, *Parabacteroides distasonis*, and *A. shahii* showed positive genetically predicted associations with increased risks of AOSD, whereas *Verrucomicrobia* and *Akkermansia* exhibited inverse associations. Mediation analysis revealed that several T-cell-related immune traits, including CD127^+^CD4^+^T cells and CD25^hi^CD4^+^T cells, partially mediated these associations, with mediation proportions ranging from 3.44% to 26.79%. Importantly, administration of *P. goldsteinii* and *A. shahii* significantly increased circulating levels of IL-1β, IL-6, and TNF-α in CIA mice, supporting the biological plausibility of the MR findings.

**Conclusion:**

This study integrates genetic and experimental evidence to support potential roles of gut microbiota in AOSD susceptibility and immune dysregulation. Specific taxa, including *P. goldsteinii* and *A. shahii*, may exhibit pro-inflammatory effects, while *Akkermansia* may have protective effects. These findings offer novel insights into microbiota-immune interactions in AOSD, and highlight potential microbial and immunological targets for future therapeutic intervention.

## 1 Introduction

Adult-onset Still’s disease (AOSD) is a rare systemic autoinflammatory disorder characterized by excessive and dysregulated inflammatory responses, typically manifesting as high fever, skin rash, systemic inflammation, and polyarthritis or diffuse arthritis (Bywaters, 1971). Unlike vulgaris arthritis, AOSD is associated with substantial morbidity and mortality, particularly in patients complicated by macrophage activation syndrome (MAS), a life-threatening hyperinflammatory syndrome driven by excessive immune activation (Crayne et al., 2019). Despite extensive investigation of its potential pathophysiological mechanisms, the etiology of AOSD remains elusive.

Current evidence suggests that AOSD arises from aberrant immune activation triggered by a combination of genetic predisposition and environmental factors (Lerner et al., 2015). Genome-wide association study (GWAS) have identified several genetic polymorphisms, particularly within the human leukocyte antigen (HLA) region, highlighting a genetic contribution to AOSD susceptibility (Wouters et al., 1986). Immunologically, AOSD is characterized by prominent activation of innate immunity, which contributes to recurrent fever and systemic inflammation, followed by adaptive immune responses that contribute to persistent arthritis (Nigrovic, 2014). Emerging evidence further indicates that the gut microbiota plays a regulatory role in modulating immune responses in autoimmune disorders (Belkaid and Hand, 2014). Gut microorganisms contribute to the maintenance of intestinal barrier integrity, modulation of metabolic pathways, and regulation of both innate and adaptive immunity, as observed in diseases like rheumatoid arthritis (RA) (Liu et al., 2024), Sjogren’s syndrome (Wang et al., 2022), systemic lupus erythematosus (SLE) (Hevia et al., 2014), and so on. These findings imply that gut microbial dysbiosis may promote abnormal immune responses and contribute to AOSD susceptibility. However, the potential genetically predicted associations between microbiota dysfunction and AOSD susceptibility remain unclear.

Mendelian randomization (MR) is a powerful genetic epidemiological approach that uses genetic variants as instrumental variables (IVs) to infer potential genetically predicted associations between exposures and disease outcomes (Davies et al., 2018). Compared with conventional observational studies, MR analysis significantly reduces biases arising from residual confounding and reverse interference, thereby providing more robust evidence for potential genetically predicted associations. In parallel, accumulating evidences have highlighted the central role of immune dysregulation in AOSD susceptibility, involving multiple immune-cell subsets and cytokine signaling pathways, particularly those mediated by interleukin-1/6 (IL-1/6) and interferons (IFNs) (Feist et al., 2018). By comprehensively integrating 731 immune-cell traits, we can identify specific immune-cell subsets and immune-related traits as potential mediators between gut microbial dysbiosis and the onset or development of AOSD.

In the present study, we aimed to systematically investigate the potential risk and protective associations between gut microbiota and AOSD susceptibility, using immune modulation as potential mediators. By integrating genetic epidemiological analyses with a comprehensive assessment of 731 immune-cell traits, this study tends to screen pivotal gut microbiota taxa and immune-cell traits potentially involved in AOSD susceptibility and to evaluate their potential as therapeutic targets. These findings may provide novel insights into the microbiota-immune axis underlying AOSD and facilitate the identification of promising therapeutic targets and precision intervention strategies for this complex autoinflammatory disease.

## 2 Methods

### 2.1 Study design

In this study, a two-sample MR approach was applied to investigate the potential associations between gut microbiota and the risks of AOSD. To further understand the mediation induced by immune-cell traits, we employed a two-step MR strategy to calculate mediation effects and validated the proinflammatory effects of critical microbiota on AOSD *in vivo*. The study’s design and analytical workflow are summarized in Figure 1.

**FIGURE 1:**
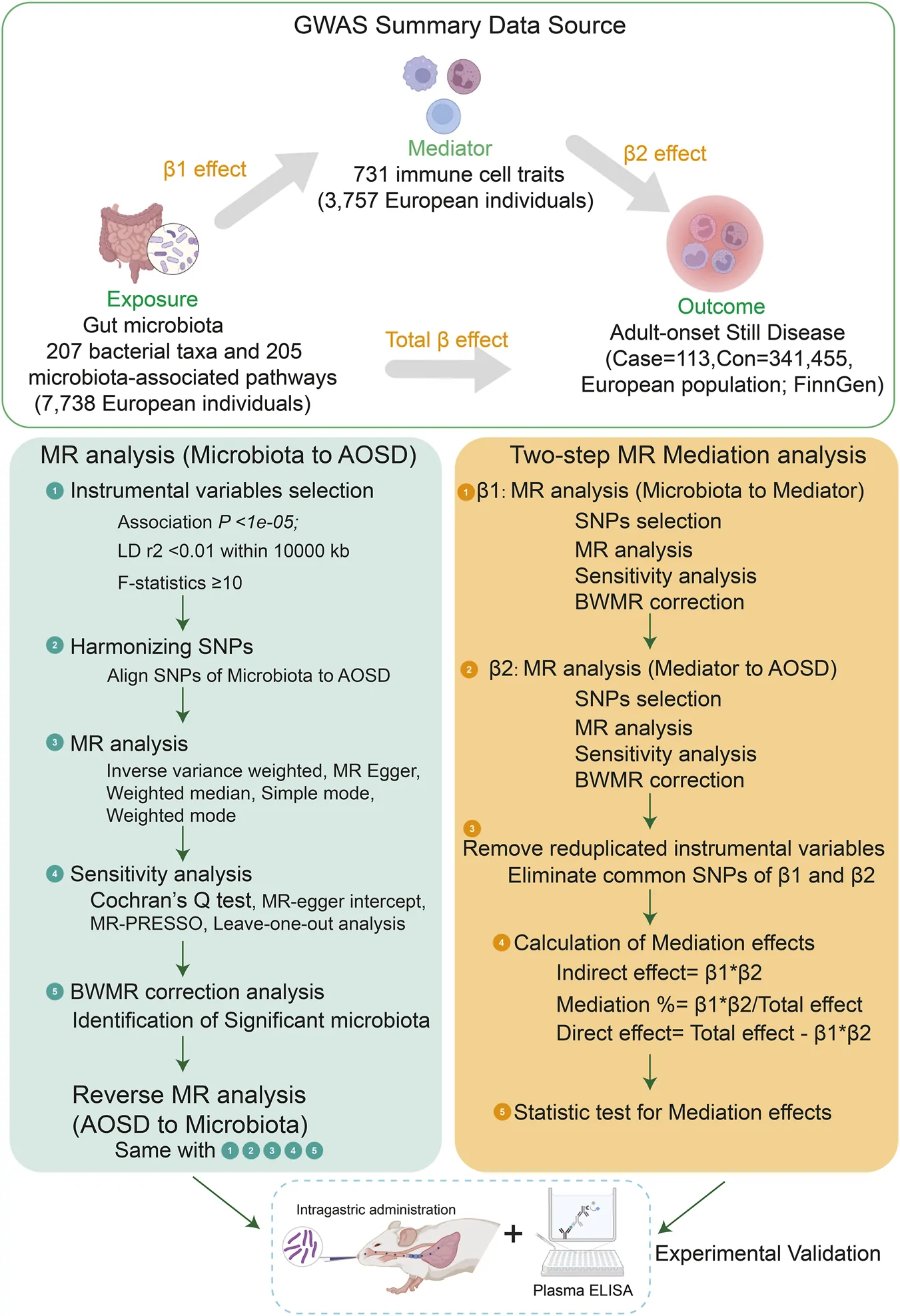
The work flow of the analytical framework in this study.

### 2.2 GWAS summary statistic datasets of exposure, mediator, and outcome

GWAS summary datasets for various gut microbiota exposures were primarily retrieved from the Dutch Microbiome Project (GWAS Catalog database: https://www.ebi.ac.uk/gwas/home, ID: GCST90027446 to GCST90027857). This large-scale GWAS study included 7,738 European participants, encompassing 207 bacterial taxa (5 phyla, 10 classes, 13 orders, 26 families, 48 genera, and 105 species) along with 205 special microbiota-associated pathways (Lopera-Maya et al., 2022). The GWAS summary statistics for AOSD patients were retrieved from the FinnGen database (https://finngen.gitbook.io/documentation, R10), involving 113 cases and 341,455 controls of European population. To further explore the mediation effects of diverse immune modulation between gut microbiota and AOSD outcomes, we incorporated another large-scale GWAS study, comprising of 731 immune cell traits derived from 3,757 European participants (GWAS Catalog database, ID: GCST90001410 to GCST90002121) (Orru et al., 2020). Comprehensive details of all data sources were provided in Supplementary Table S1.

### 2.3 Instrumental variables selection

The MR analysis is constructed based on three fundamental core assumptions, including independent IVs from confounders, strong associations between IVs and exposures, and inevitability of IVs’ effects on outcomes via exposures. Hence, we initially identified single nucleotide polymorphisms (SNPs) linked to exposures with potentially genetic associations (*P* < 1e−5). Linkage disequilibrium (LD) clumping was subsequently performed to ensure independence of the selected SNPs, using an LD threshold of r^2^ < 0.001 and a clumping window size <10,000 kb. Subsequently, palindromic SNPs with allele frequencies close to 0.5 were excluded to minimize strand ambiguity, and harmonization procedures were applied to align exposure and outcome datasets. To evaluate instrument strength and minimize weak-instrument bias, F statistics were calculated using the following formula: $F=\frac{R^{2}}{1-R^{2}}*\frac{N-K-1}{K}$ , where R^2^ represents the total variance of exposures explained by selected SNPs, N represents the sample size, and K represents the number of chosen IVs. The SNPs with F-value ≥10 were considered to have adequate instrument strength, thereby ameliorating the limitations of weak instrument bias in the two-sample MR method. Given the relatively limited number of AOSD cases in the FinnGen cohort, additional measures were implemented to enhance the reliability and robustness of the MR analysis. Specifically, stringent SNP-selection criteria and LD pruning were applied to reduce potential bias arising from weak or correlated instruments. Furthermore, multiple complementary MR methods and sensitivity analyses were performed to assess the consistency and stability of the genetically predicted estimates, thereby reducing the likelihood that the observed associations resulted from unstable instrumental effects or statistical artifacts associated with limited case numbers.

### 2.4 MR and sensitivity analysis

Bidirectional two-sample MR analysis was performed to explore the potential risk relationships between various microbiota taxa and AOSD susceptibility. Potentially pleiotropic IVs were identified using the PhenoScanner database and excluded if they were associated with known confounders or the AOSD outcome (*P* < 0.01). The primary evaluation of MR results was conducted using the inverse-variance weighted (IVW) method, complemented with estimations from MR‐Egger, weighted median (WM), Simple mode, and Weighted mode methods, using the TwoSampleMR package (https://github.com/MRCIEU/TwoSampleMR) (Burgess et al., 2013). For sensitivity analysis, heterogeneity among the instrumental estimates was assessed using Cochran’s Q test, with *P* > 0.05 indicating no statistically significant heterogeneity (Kulinskaya and Hoaglin, 2023). Horizontal pleiotropy was evaluated using the intercept from MR-Egger regression (Bao et al., 2023) and the global test of the MR‐pleiotropy residual sum and outlier (MR-PRESSO) test (Verbanck et al., 2018), with *P* > 0.05 indicating no evidence of statistically significant horizontal pleiotropy. To further enhance the robustness of our findings, the analyses were independently repeated using the Bayesian weighted Mendelian randomization (BWMR) method, and only microbiota with significance after BH correction (BH-adjusted p-values <0.05) in both IVW and BWMR analyses were identified as having potential genetically predicted associations with AOSD susceptibility.

### 2.5 Mediation analysis

To investigate whether immune-cell traits mediated the potential associations of gut microbiota on AOSD, a two-step MR mediation framework was applied. First, reverse MR analyses were conducted using AOSD as the exposure and the identified gut microbiota taxa or microbial pathways as outcomes to exclude potential reverse influence. Taxa and pathways showing significant reverse genetically predicted associations were removed from subsequent mediation analyses. Subsequently, a two-step mediation analysis was performed. In the first step, MR analyses were conducted between gut microbiota (exposure) and immune-cell traits (mediators), generating the potential genetically predicted effects (β_1_). In the second step, MR analyses were performed between immune-cell traits and AOSD, yielding the potential genetically predicted effects (β_2_). Only immune-cell traits that remained significant after BH correction in both microbiota-to-immune-cell and immune-cell-to-AOSD analyses, and demonstrated consistent effect directions across IVW and BWMR methods, were retained as candidate mediators. Immune-cell subsets that failed to achieve significance in either step or exhibited inconsistent effect directions were excluded from the final mediation analysis.

The total genetically predicted effect (β_3_) was defined as the overall effect of gut microbiota on AOSD obtained from the primary univariable MR analysis. The indirect (mediated) effect was calculated as: $\text{Indirect effect}=\beta_{1}\times \beta_{2}$ and the mediation proportion was estimated as: Mediation proportion $=\frac{\beta_{1}\times \beta_{2}}{\beta_{3}}$ . The direct effect, representing the remaining effect of gut microbiota on AOSD independent of immune-cell mediation, was calculated as: $\text{Direct effect}=\beta 3‐\left(\beta 1\times \beta 2\right)$ . Here, the indirect effect reflects the proportion of the microbiota-AOSD association mediated through immune-cell traits, whereas the direct effect represents the residual effects not explained by the selected mediators. All mediation analyses were subjected to rigorous statistical evaluation to ensure the robustness and validity of the findings.

### 2.6 Cultivation of *Parabacteroides goldsteinii* (*P. goldsteinii, PG*) and *Alistipes shahii* (*A. shahii, AS*)

*Parabacteroides goldsteinii* (BNCC342457) and *A. shahii* (BNCC354336) were purchased from the BeNa Culture Collection. Both strains were cultivated under strictly anaerobic conditions using Columbia blood plate (CA-B) supplemented with liquid thioglycollate medium in an anaerobic workstation (DG250; Don Whitley Scientific, United Kingdom). The chamber atmosphere was maintained at 37 °C and consisted of a gas mixture containing 5% CO_2_, 5% H_2_, and 90% N_2_. Following incubation, bacterial cells were harvested and pelleted by centrifugation, then resuspended in sterile saline to achieve a final concentration of 5 × 10^9^ colony-forming units (CFU), which was used for subsequent experimental applications.

### 2.7 Animal experiments

A total of 30 specific pathogen-free (SPF) C57BL/6 mice (6 weeks of age) were obtained from Charles River Japan Inc. and randomly allocated into control and collagen-induced arthritis (CIA) groups. Although the CIA model reproduces several key inflammatory and systemic manifestations relevant to AOSD, including excessive systemic cytokine activation, innate and adaptive immune dysregulation, splenomegaly, and severe inflammatory arthritis (Koga et al., 2023), it was established as a classical murine model of rheumatoid arthritis rather than a disease-specific model of AOSD. Therefore, the experimental studies were designed to evaluate the pro-inflammatory effects of candidate microbial taxa and to provide biological support for the inflammatory plausibility of the MR findings, rather than to directly validate AOSD pathogenesis.

To induce CIA, mice at 8 weeks of age were immunized on day-0 with 100 μg of type II chicken collagen (Creative Enzymes, United States) emulsified at a 1:1 ratio with complete Freund’s adjuvant (CFA; Creative Enzymes, United States), followed by a booster injection with the same emulsion on day-21. Control mice received equal volumes of phosphate-buffered saline (PBS) emulsified with incomplete adjuvant under identical experimental conditions. Both control and CIA mice were further subdivided into microbiota intervention groups and corresponding vehicle-treated groups. Prior to bacterial colonization, mice received a broad-spectrum antibiotic cocktail in drinking water *ad libitum* for 14 consecutive days to establish a relatively microbiota-depleted intestinal environment. The antibiotic cocktail consisted of ampicillin sodium (1.0 g/L), gentamicin sulfate (1.0 g/L), vancomycin hydrochloride (0.5 g/L), and imipenem (0.25 g/L). Antibiotic-containing water was freshly prepared every 2 days to maintain antimicrobial activity, and cages were randomly allocated to antibiotic-treated or vehicle-treated conditions to minimize environmental bias. Following this pretreatment, control mice received PBS via oral gavage, whereas experimental groups were administered either *P. goldsteinii* or *A. shahii* at a dose of 5 × 10^9^ CFU/day for 14 consecutive days.

### 2.8 Experimental support of AOSD-related inflammation via enzyme-linked immunosorbent assay (ELISA)

Peripheral blood was obtained from each mouse, and plasma levels of inflammatory cytokines were quantified by ELISA. The concentrations of interleukin-1β (IL-1β), interleukin-6 (IL-6), and tumor necrosis factor-α (TNF-α) were measured using commercially available 96-well plates pre-coated with capture antibodies (Arigo Biolaboratories Corp, Taiwan, China). Calibration curves were established by serial dilution of a 150 μL stock standard to generate five graded concentrations. For each assay, 50 μL of plasma sample and 50 μL of biotin-conjugated detection reagent were added to each well and incubated at 37 °C for 60 min. Following five washing cycles to remove unbound components, 50 μL of avidin–horseradish peroxidase conjugate was added and incubated for an additional 30 min. Subsequently, chromogenic substrates A and B were introduced and allowed to react for 10 min in the dark, after which the enzymatic reaction was terminated with stop solution. Optical density was recorded at 450 nm, and cytokine concentrations were calculated using ELISAcalc based on a logistic regression fitting model.

### 2.9 Statistical analysis

All statistical analyses were performed using R software (version 4.3.2). Mendelian randomization analyses were conducted using the “TwoSampleMR”, “BWMR”, and “MR-PRESSO” packages. The IVW method was used as the primary analytical approach, while MR-Egger, weighted median, simple mode, weighted mode, and BWMR methods were applied for validation and sensitivity analyses. Heterogeneity and horizontal pleiotropy were assessed using Cochran’s Q test, MR-Egger intercept analysis, and MR-PRESSO. For animal experiments, data were presented as mean ± standard deviation (SD), and differences between groups were evaluated using Wilcoxon’s test. To account for the substantial multiple-testing burden across different analytical stages, false discovery rate (FDR) correction with Benjamini-Hochberg (BH) methods was applied separately within each analysis category. Specifically, FDR correction was performed independently for: (i) the 412 gut microbiota exposures (207 taxa and 205 microbial pathways) evaluated in the microbiota-AOSD MR analyses; (ii) the 731 immune-cell traits examined in the immune cell-AOSD MR analyses; and (iii) the microbiota/pathway-immune-cell MR analyses conducted during mediator screening. Candidate immune-cell mediators were retained only when they achieved significance after BH correction in both the IVW and BWMR analyses and showed consistently predicted directions. For mediation analyses, only microbiota-immune-cell pairs that remained significant after FDR correction in the preceding screening steps were carried forward. Because mediation analyses were hypothesis-driven and restricted to a limited number of preselected exposure-mediator pairs, nominal *P*-values are reported together with mediation proportions. Throughout the manuscript, BH-adjusted *P-*values are reported when multiple-testing correction was applied, whereas nominal *P*-values are explicitly indicated without BH correction where appropriate.

## 3 Results

### 3.1 Two-sample MR analysis between gut microbiota and AOSD outcomes

After BH correction for 412 microbiota-related exposures, 13 associations remained statistically significant (BH-adjusted *P* < 0.05), including 10 microbial taxa and 3 microbial metabolic pathways. All findings reported below refer to associations that remained significant after multiple-testing correction unless otherwise stated. (Figure 2). These findings included significant potential associations of Family Streptococcaceae (OR: 2.66; 95% CI: 1.37–5.17, BH-adjusted *P* = 0.019), Species *Alistipes shahii* (OR: 3.22; 95% CI: 1.03–10.10, BH-adjusted *P* = 0.045), Genus *Streptococcus* (OR: 3.48; 95% CI: 1.52–7.99, BH-adjusted *P* = 0.019), Species *Parabacteroides distasonis* (OR: 3.94; 95% CI: 1.39–11.21, BH-adjusted *P* = 0.028), Species *Parabacteroides goldsteinii* (OR: 2.34; 95% CI: 1.28–4.28, BH-adjusted *P* = 0.019), Creatinine degradation (OR: 4.05; 95% CI: 1.53–10.72, BH-adjusted *P* = 0.019), and L-tryptophan biosynthesis (OR: 3.62; 95% CI: 1.13–11.58, BH-adjusted *P* = 0.043) with increased risks in AOSD outcomes. In contrast, several taxa and pathways displayed significant protective roles in the genetic predisposition to AOSD, including *Verrucomicrobiae* at Class, Family, Genus and Phylum levels (OR: 0.33; 95% CI: 0.12–0.92, BH-adjusted *P* = 0.043), Genus *Akkermansia* (OR: 0.33; 95% CI: 0.12–0.92, BH-adjusted *P* = 0.045), and Mannan degradation (OR: 0.28; 95% CI: 0.08–0.96, BH-adjusted *P* = 0.048). Notably, the sensitivity analysis indicated that heterogeneity existed in Genus *Akkermansia* and *Verrucomicrobia* at Class, Family, Genus and Phylum levels, but no pleiotropy existing in all 13 taxa and pathways (Supplementary Table 2). Moreover, no statistically significant association was observed in the reverse MR analysis for all 13 taxa and pathways, confirming their appropriateness for subsequent mediation analysis (nominal *P* > 0.05, Table. 1).

**FIGURE 2:**
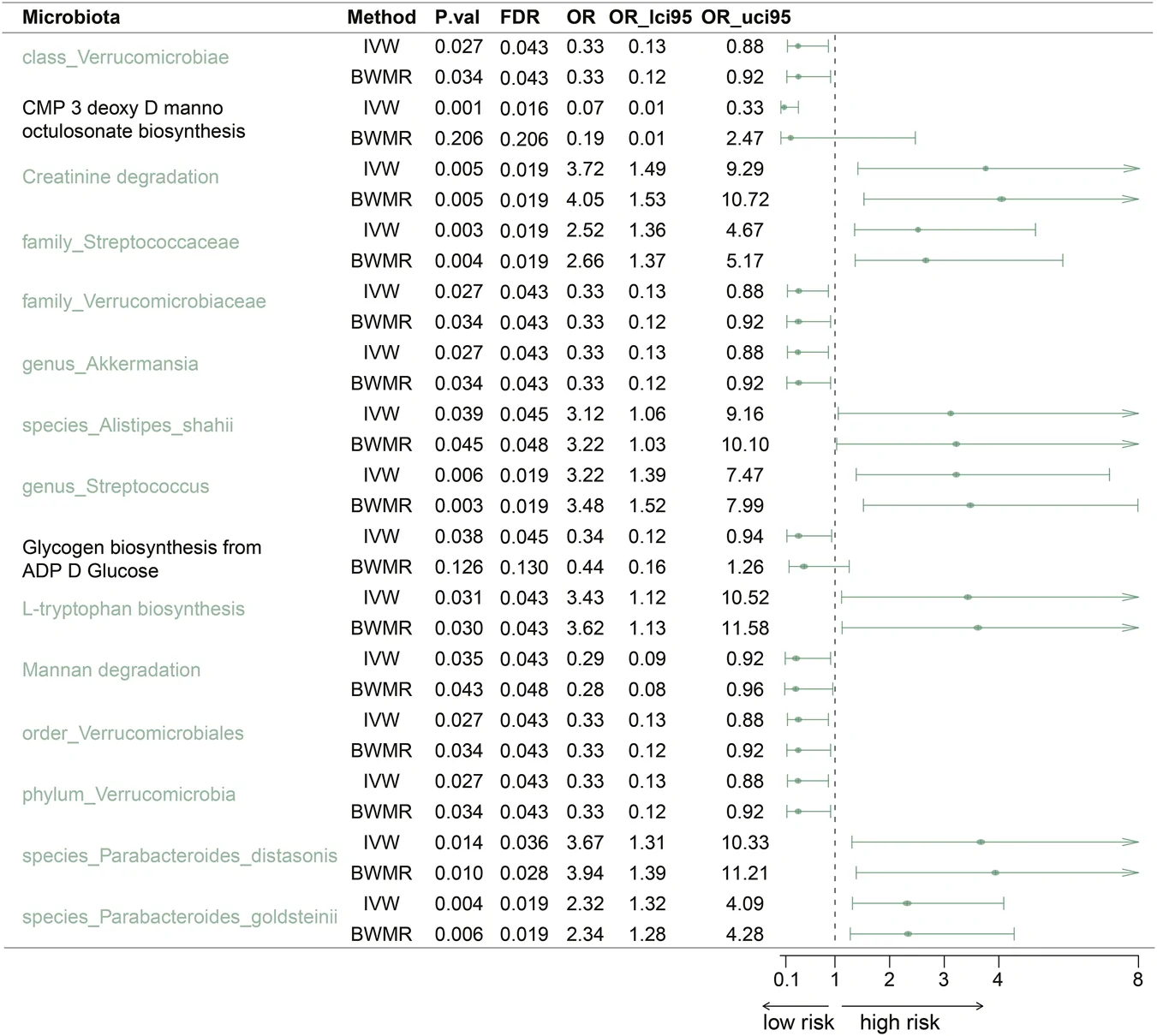
The MR results for the association between gut microbiota and AOSD. The significant microbiota was identified as BH-adjusted *P*_IVW_ and BH-adjusted *P*_BWMR_ < 0.05, and the forest diagram exhibited the odds ratio (OR) and 95% confidence intervals (CI) for the microbiota exposures.

**TABLE 1:** Reverse MR results of AOSD traits and significant gut microbiota outcomes.

Exposures	Outcomes/microbiota	Method	β value	SE	Nominal *P-*value	OR [95% CI]
AOSD	Creatinine degradation	IVW	0.02	0.01	0.184	1.02 [0.99–1.04]
AOSD	Glycogen biosynthesis from ADP D glucose	IVW	0.01	0.01	0.245	1.01 [0.99–1.03]
AOSD	CMP 3 deoxy D manno octulosonate biosynthesis	IVW	0.003	0.01	0.750	1.003 [0.98–1.02]
AOSD	Mannan degradation	IVW	−0.02	0.01	0.056	0.98 [0.96–1.00]
AOSD	L tryptophan biosynthesis	IVW	0.004	0.01	0.671	1.004 [0.98–1.03]
AOSD	class_*Verrucomicrobiae*	IVW	−0.01	0.02	0.457	0.99 [0.95–1.02]
AOSD	family_*Streptococcaceae*	IVW	−0.02	0.01	0.173	0.98 [0.96–1.01]
AOSD	family_*Verrucomicrobiaceae*	IVW	−0.01	0.02	0.457	0.99 [0.95–1.02]
AOSD	genus_*Streptococcus*	IVW	−0.02	0.01	0.209	0.98 [0.96–1.01]
AOSD	genus_*Akkermansia*	IVW	−0.01	0.02	0.457	0.99 [0.95–1.02]
AOSD	order_*Verrucomicrobiales*	IVW	−0.01	0.02	0.457	0.99 [0.95–1.02]
AOSD	phylum_V*errucomicrobia*	IVW	−0.01	0.02	0.457	0.99 [0.95–1.02]
AOSD	species_*Parabacteroides_distasonis*	IVW	0.001	0.01	0.913	1.001 [0.98–1.02]
AOSD	species_*Parabacteroides_goldsteinii*	IVW	−0.02	0.02	0.229	0.98 [0.94–1.02]
AOSD	species_*Alistipes_shahii*	IVW	−0.01	0.01	0.644	0.99 [0.97–1.02]

AOSD, Adult-onset Still disease; IVW, inverse-variance weighted; SE, standard error; CI, confidence interval.

### 3.2 Identification of significant mediators

To screen significant mediators, we examined the potential associations between 731 immune cell traits and AOSD, and successfully pinpointed several significant associations (Figure 3). Among the 731 immune-cell traits evaluated, 28 showed nominal significance in the IVW analysis (nominal *P* < 0.05). Following BH correction, 20 immune-cell traits remained statistically significant (BH-adjusted *P* < 0.05) and were subsequently retained for mediator screening. Multiple immune cells exhibited potential risk for AOSD, including CD25 on CD39^+^CD4^+^T cell (OR: 1.31; 95% CI: 1.03–1.65, BH-adjusted *P* = 0.043), CD25^hi^ CD45RA^+^ CD4 not Treg AC (OR: 1.38; 95% CI: 1.10–1.72, BH-adjusted *P* = 0.028), CD45 on basophil (OR: 1.54; 95% CI: 1.10–2.15, BH-adjusted *P* = 0.035), CD8 on NKT (OR: 1.59; 95% CI: 1.03–2.47, BH-adjusted *P* = 0.048), IgD-CD38br %B cell (OR: 1.85; 95% CI: 1.13–3.04, BH-adjusted *P* = 0.035), and Transitional AC (OR: 1.48; 95% CI: 1.02–2.80, BH-adjusted *P* = 0.043). Conversely, the other immune cells displayed significant protective roles in AOSD, particularly in T-lymphocyte related subsets, such as CD127 on CD4^+^ T (OR: 0.50; 95% CI: 0.27–0.92, BH-adjusted *P* = 0.043), CD28^+^ DN (CD4^−^CD8^−^) %T cell (OR: 0.66; 95% CI: 0.45–0.98, BH-adjusted *P* = 0.043), CD4 on EM CD4^+^T (OR: 0.63; 95% CI: 0.47–0.86, BH-adjusted *P* = 0.024), and so on (Figure 3). Similarly, no heterogeneity and pleiotropy were found in these immune cells (Supplementary Table S3).

**FIGURE 3:**
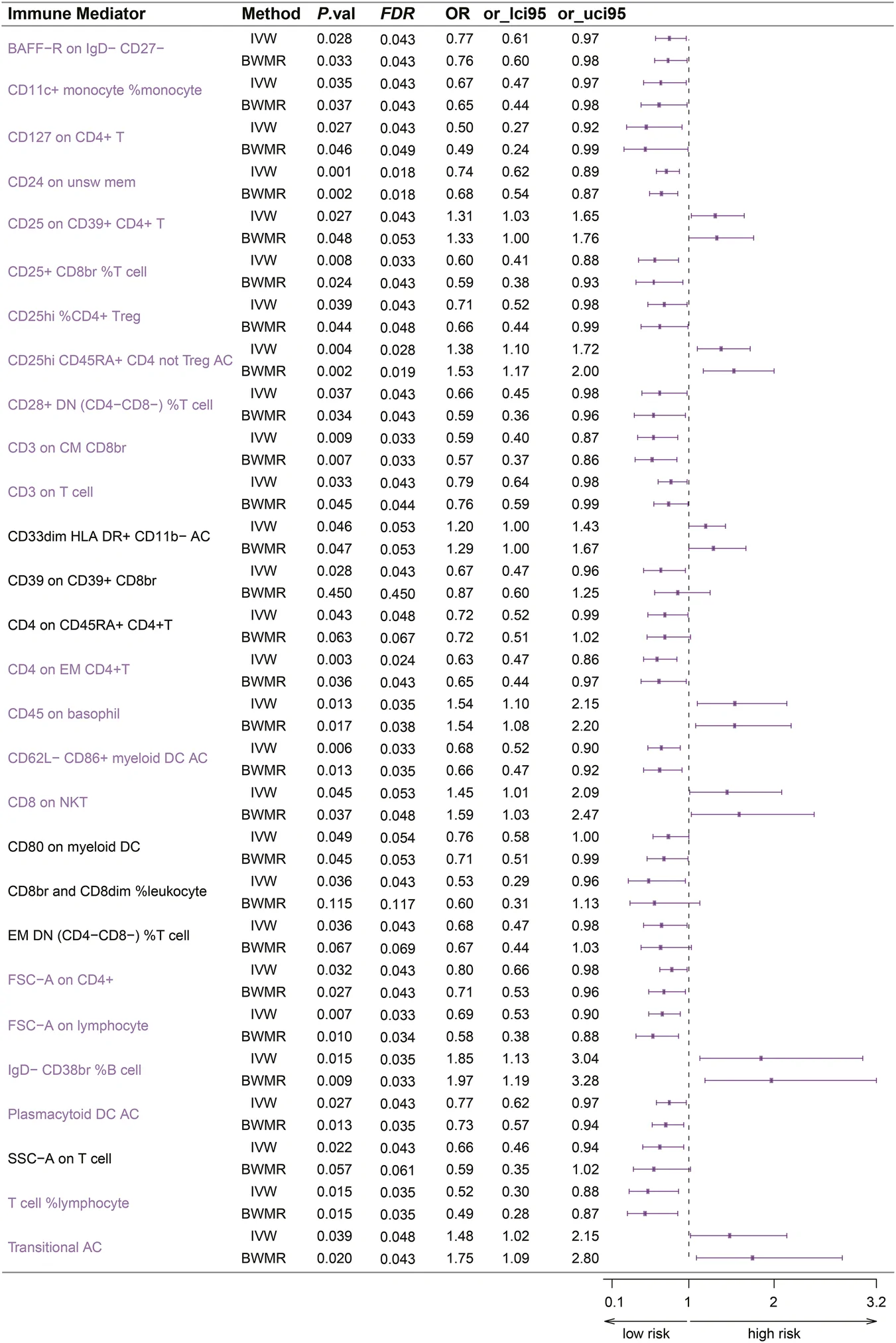
The MR results for the association between immune-cell traits and AOSD. The significant immune-cell mediator was identified as BH-adjusted *P*_IVW_ and BH-adjusted *P*_BWMR_ < 0.05, and the forest diagram exhibited the odds ratio (OR) and 95% confidence intervals (CI) for the immune-cell exposures.

Following our exploration of the effects of immune cell traits on AOSD, we further investigated the potential mediation effects of significant gut microbiota exposures on above significant immune cells (Figure 4). After the BH correction with BWMR analysis, we successfully detected three positive pathway-cell and five negative microbiota-cell mediations (Supplementary Table S4). The sensitivity analysis demonstrated the absence of heterogeneity and pleiotropy in those potential relationships (Supplementary Figure S1). The Family Streptococcaceae exhibited inhibitory roles in T cell %lymphocyte (OR: 0.84; 95% CI: 0.72–0.98, BH-adjusted *P* = 0.034), and Species *A. shahii* displayed inhibitory effects on CD3 on CM CD8^+^ T cell and CD25hi CD45RA^+^ CD4 not Treg AC (OR: 0.69; BH-adjusted *P* = 0.029; OR: 0.70; BH-adjusted *P* = 0.027, respectively). Additionally, Species *P. goldsteinii* was negatively associated with CD25^hi^CD45RA^+^CD4 not Treg AC (OR: 0.75; 95% CI: 0.59–0.95, BH-adjusted *P* = 0.027) and CD127 on CD4^+^T cell (OR: 0.72; 95% CI: 0.55–0.93, BH-adjusted *P* = 0.027). Interestingly, Creatinine degradation showed significant positive-association with FSC-A on lymphocyte (OR: 1.39; BH-adjusted *P* = 0.027) and FSC-A on CD4^+^ T cell (OR: 1.42; BH-adjusted *P* = 0.031), and L-tryptophan biosynthesis exhibited significant stimulative effects on CD25^hi^CD45RA^+^CD4 not Treg AC (OR: 1.34; BH-adjusted *P* = 0.039) (Figure 4b) These findings indicated that immune-cell traits may partially mediate the association between gut microbiota/pathway and AOSD outcomes.

**FIGURE 4:**
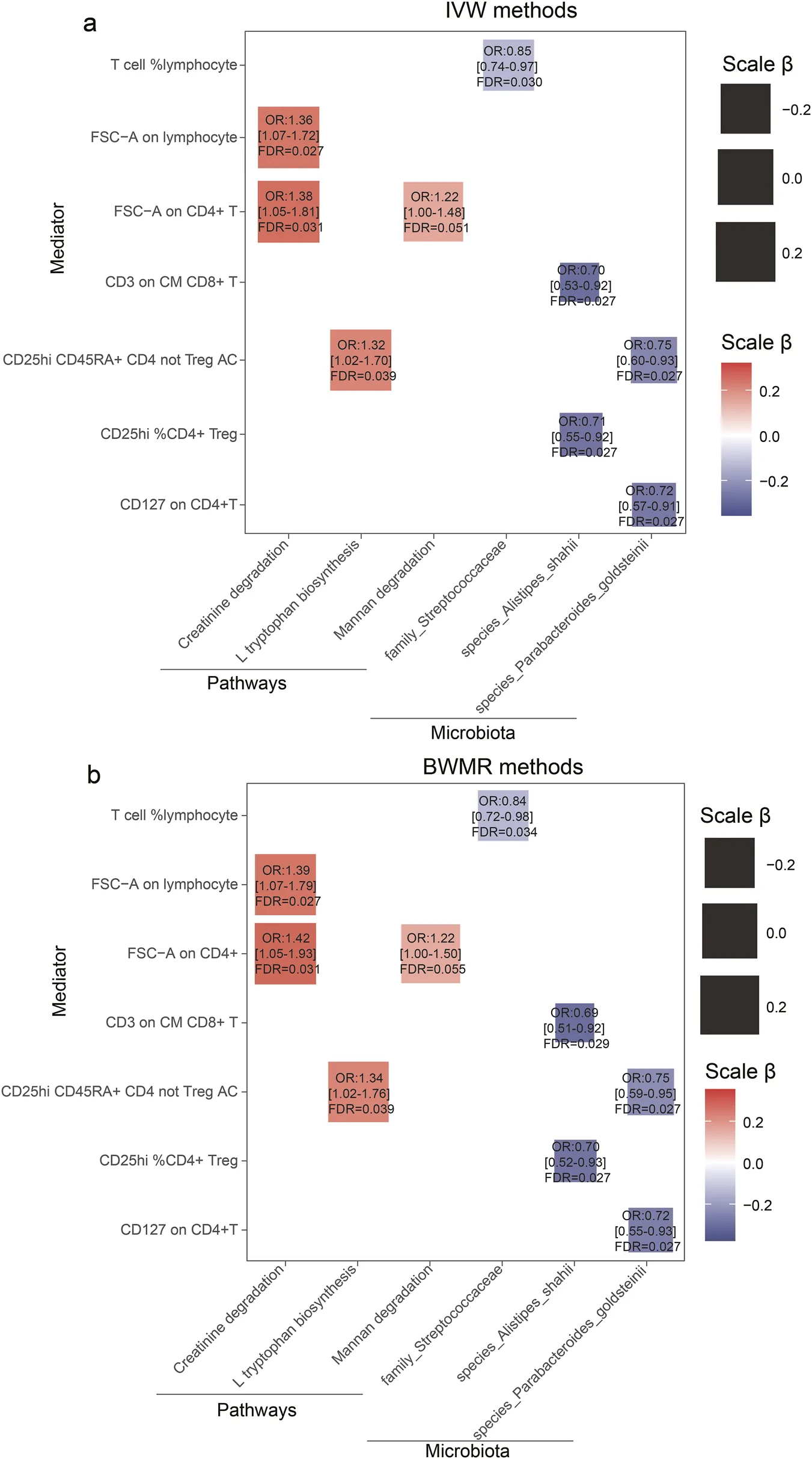
The MR results for the association between gut microbiota/pathways and immune-cell mediators. The results were obtained by IVW **(a)** and BWMR methods **(b)**. The red represents risk associations and blue represents protective effects. The size and depth of block represent β values.

### 3.3 Evaluation of mediation analysis

Through integrating the mediation effects from eight regulatory associations, we ultimately identified six reasonable mediated regulations for the potential associations of bacterial taxa and pathways on risks of AOSD. The Family Streptococcaceae mediated its effect on AOSD through T lymphocyte with proportions of 11.94% (nominal *P* = 0.028), and Species *P. goldsteinii* exhibited mediation effects via CD127 on CD4+T cell with proportions of 26.79% (nominal *P* = 0.028) (Figures 5a,b). Furthermore, the Species *A. shahii* channeled its potential contributors through CD25^hi^CD4^+^Treg (9.96%, nominal *P* = 0.020), and CD3 on CM CD8^+^T cell (16.48%, nominal *P* = 0.010) (Figures 5c,d). For the metabolic processes, Mannan degradation exhibited its protective associations through FSC-A on CD4^+^T cells with mediated proportions of 3.44% (nominal *P* = 0.049), while CD25^hi^ CD45RA^+^ CD4 not Treg AC displayed 7.20% mediation effects in the risk effects of L-tryptophan biosynthesis (nominal *P* = 0.035) (Supplementary Figures S2a,b). There was no mediation effect existing in the regulations of Creatinine degradation and Species *P. goldsteinii* (Supplementary Figures S2c–e). These proportions displayed the intricate dynamics among specific microbiota exposures, pivotal mediators, and corresponding cumulative effects on AOSD.

**FIGURE 5:**
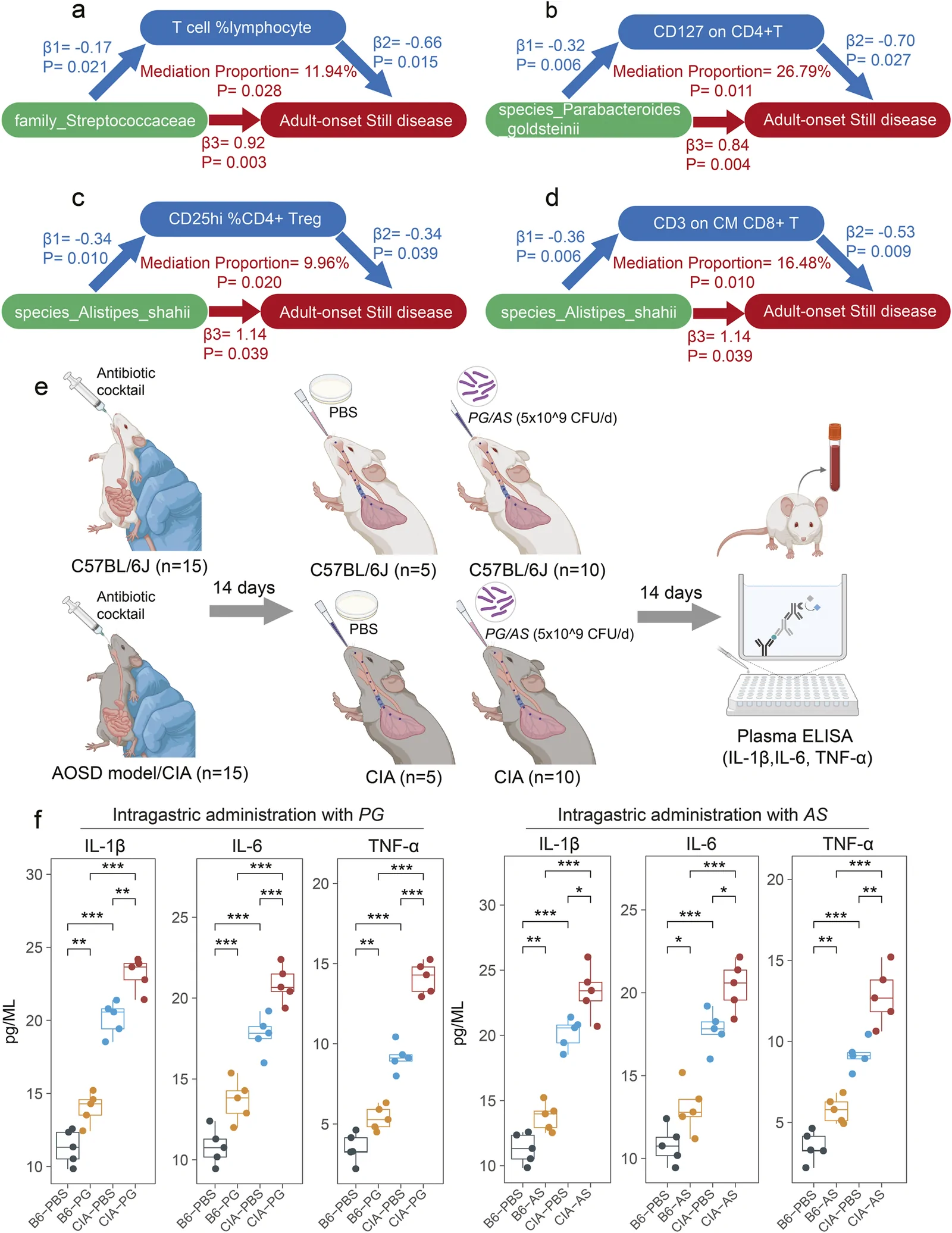
Mediation analysis and *in vivo* biological support. **(a–d)** Mediation analysis of immune-cell trait between significant gut microbiota and AOSD. The red represents risk associations and blue represents protective effects. **(e)** The experimental workflow for establishing a relatively microbiota-depleted murine model followed by PBS or microbial intervention. **(f)** CIA mice subjected to microbial intervention demonstrated the highest concentrations of all three pro-inflammatory cytokines in both bacterial treatment subgroups. *, *P* < 0.05; **, *P* < 0.01; ***, *P* < 0.001.

### 3.4 Experimental support of pro-inflammatory effects of *P. goldsteinii* and *A. shahii in vivo*

The experimental workflow for establishing a relatively microbiota-depleted murine model followed by PBS or microbial intervention is presented in Figure 5e. Upon intragastric administration of *P*. *goldsteinii* and *A*. *shahii*, mice receiving microbiota supplementation exhibited significantly elevated circulating levels of IL-1β, IL-6, and TNF-α compared with PBS-treated controls in both C57BL/6 and CIA groups. Notably, CIA mice subjected to microbial intervention demonstrated the highest concentrations of all three pro-inflammatory cytokines in both bacterial treatment subgroups. These findings collectively support the pro-inflammatory properties of *P. goldsteinii* and *A. shahii* and suggest their biological plausibility in the potential associations with the risk of AOSD (Figure 5f).

## 4 Discussion

The intricate connection between gut microbial dysbiosis and immune-mediated diseases has attracted considerable attention in recent years. The microbiome and immune system interact extensively to maintain physiological homeostasis, and disruption of this balance may contribute to immune-mediated disease (Xu et al., 2019; Zheng et al., 2020). Our study explored the potential genetically predicted associations between specific microbiota taxa and AOSD through MR analysis, providing promising insights into the microbiota-immune axis underlying AOSD. The *Verrucomicrobia (phylum), Verrucomicrobiae (class), Verrucomicrobiales (order),* Verrucomicrobiaceae *(family)*, and *Akkermansia (genus)* were inversely associated with AOSD risk, suggesting that genetically predicted higher abundance of these taxa may be associated with a lower susceptibility to AOSD. Conversely, the Streptococcaceae *(family), Streptococcus (genus), A. shahii (species), P. goldsteinii (species)*, and *P. distasonis (species)* were positively associated with AOSD risk, indicating that these taxa might have potential associations with increased susceptibility to the disease.

The human microbiota comprises trillions of microorganisms, including various bacteria, fungi, and other infecting viruses (Ursell et al., 2012). Microbial dysbiosis, characterized by compositional or functional abnormalities in the microbiome, has been implicated in the pathogenesis of various autoimmune and inflammatory diseases (Li et al., 2018). For example, several specific microbial taxa have been implicated in the initiation and progression of RA, including *Prevotella copri*, *Aggregatibacter actinomycetemcomitans*, and *Porphyromonas gingivalis* (Scher et al., 2013; Konig et al., 2016). In addition, metagenomic next-generation sequencing (mNGS) has revealed significant enrichment of the genera *Parainfluenzae, Ralstonia*, and *Aspergillus* in blood samples from AOSD patients (Fang et al.). Similarly, patients with Sjögren’s syndrome have been reported to exhibit reduced abundances of several commensal genera (i.e., *Bacteroides*, *Prevotella*, *Parabacteroides*, and *Faecalibacterium*), together with increased abundances of potentially disease-associated bacteria (i.e., *Pseudobutyrivibrio*, *Streptococcus*, and *Escherichia*), which were also positively associated with disease activity (Rusthen et al., 2019). All of these studies underscore the significant connection between gut microbiota and multiple autoimmune diseases.

Belonging to the Verrucomicrobiaceae (family), *Akkermansia* (genus) is a Gram-negative and oxygen-tolerant anaerobic bacterium that is considered as commensal microbiota in health cohorts (Ottman et al., 2016). *Akkermansia* predominantly resides in intestines and accounts for approximately 1%–4% of the total gut microbiota, where its abundance is essential for intestinal homeostasis (Derrien et al., 2008). In SLE patients, *Akkermansia* has been implicated in maintaining gut barrier integrity and mucosal immune homeostasis, and its abundance exhibits significant negative correlations with serum complement levels, suggesting potential protective effects in SLE (Liu et al., 2021). Consistent with these observations, our MR analysis identified inverse associations of *Akkermansia* (genus) and Verrucomicrobiaceae (family) with AOSD risk, supporting their potential protective roles. In contrast, the *P. goldsteinii* (species) and *P. distasonis* (species), both members of *Parabacteroides* (genus), have been reported to exert context-dependent effects across different autoimmune and inflammatory diseases. These microbiota taxa have demonstrated anti-inflammatory properties by strengthening the gut epithelial barrier in colitis mouse model (Cuffaro et al., 2020), while also showing proinflammatory effects in ankylosing spondylitis through bacterial peptide-induced expansion of IFN-γ-producing cells (Zhou et al., 2020). Similarly, *A. shahii* (species) has been reported to exhibit context-dependent effects across different diseases, including potentially protective effects in colitis, liver fibrosis, and cardiovascular diseases, but potentially pathogenic roles in colorectal cancer and depression (Parker et al., 2020). In the present study, genetically predicted higher abundances of *P. goldsteinii*, *distasonis*, and *A. shahii*, were positively associated with increased risks of AOSD, supporting their potential involvement in disease susceptibility. Moreover, animal experiments provided additional evidence for the pro-inflammatory properties of *P. goldsteinii* and *A. shahii* in both C57BL/6 and CIA models. Unlike the aforementioned taxa, the *Streptococcus* is a genus that includes numerous pathogenic species associated with substantial human morbidity and mortality. The complex interactions among *Streptococci*, complement activation, and anti-streptococcal response might contribute to autoimmune diseases via multiple mechanisms, including systemic toxicity, autoreactivity, and molecular mimicry (Syed et al., 2020). However, although the CIA model reproduces several inflammatory features relevant to AOSD, it remains primarily a model of inflammatory arthritis rather than a validated disease-specific model of AOSD. Therefore, the animal experiments in this study should be interpreted as complementary evidence supporting the pro-inflammatory potential of the identified microbial taxa and the biological plausibility of the MR findings. These results do not provide direct evidence for AOSD-specific pathogenic mechanisms. Accordingly, extrapolation of these findings to AOSD should be made with caution, and future studies employing disease-specific models and histopathological analyses are warranted to further elucidate the mechanistic roles of these taxa in AOSD pathogenesis.

Gut microbiota may influence the onset and progression of immune-mediated diseases via regulating immune cells or targets. Several studies have demonstrated that specific microbial species modulate inflammation responses by altering the balance of helper T (Th) cell and regulatory T (Treg) cell subsets. In mouse models, colonization with segmented filamentous bacteria promotes inflammatory responses via the induction of Th17 cells (Wu et al., 2010), whereas Clostridia-induced Treg cells suppress autoimmune responses (Atarashi et al., 2011). To further evaluate the potential mediation effects of immune cells on the potential genetically predicted associations between gut microbiota and AOSD, we performed the mediation MR analysis. The results revealed that three microbiota taxa were potentially genetically associated with AOSD susceptibility by regulating T-lymphocyte subsets. Specifically, the *Streptococcus* (family) showed mediation effects through T cell %lymphocyte, and the *P. goldsteinii* (species) exhibited mediation effects through CD127 on CD4^+^T cells. CD127/IL-7R has been recognized as a major homeostatic cytokine supporting the survival of various mature T cell populations (Carrette and Surh, 2012). Although conventional Treg cells typically exhibit low CD127 expression, its expression may increase upon Treg-cell activation and has been implicated in the regulation of immune responses (Simonetta et al., 2010). Moreover, the *A. shahii* (species) showed potential mediated effects through CD25^hi^CD4^+^Treg cell and CD3 on CD8^+^central memory T (Tcm) cells. CD8^+^ Tcm and effector memory T (Tem) cells are major circulating memory T-cell subsets, both capable of producing IL-2 and effector cytokines following antigenic stimulation. However, Tcm cells preferentially exhibit lymphoid-homing characteristics and greater proliferative capacity, whereas Tem cells are generally characterized by more pronounced effector cytokine production (Samji and Khanna, 2017). These findings indicate that specific gut-microbiota may influence genetically predicted AOSD susceptibility via modulation of adaptive immune-cell subsets, particularly Treg and Tcm cells. Nevertheless, given the modest mediation proportions observed in this study, these immune-cell traits should be interpreted as potential partial mediators rather than definitive mechanistic intermediates.

One advantage of this study is the use of a rigorous two-sample MR framework, complemented by bidirectional analyses and multiple sensitivity tests. This approach substantially minimized confounding bias and reverse genetically predicted effects, thereby strengthening the robustness of potential inference. Furthermore, the integration of large-scale multi-dimensional datasets, including gut microbiota, 731 immune-cell traits, and AOSD-related GWAS data, enabled a comprehensive and systematic exploration of the microbiota-immune-disease axis. Finally, the incorporation of two-step mediation MR analysis and *in vivo* experimental support provides both mechanistic insight and biological plausibility, enhancing the translational relevance of the findings.

However, there are still several limitations in our study. First, the study populations were restricted to individuals of European ancestry, which may limit the generalizability of the findings to other ethnic groups. Second, the use of GWAS summary-level data precluded access to individual-level clinical variables, including age, sex, and disease severity, thereby limiting more refined stratified or phenotypic analyses. Third, the FinnGen’s GWAS dataset of AOSD included only 113 cases, reflecting the rarity of AOSD but also representing a potential limitation for MR analysis. The relatively small sample size may reduce statistical power and increase the risk of false-negative findings or unstable effect estimates, particularly for associations with modest effect sizes. Given the limited number of AOSD cases, our findings should still be interpreted cautiously and require further validation in larger independent cohorts and multicenter studies. Fourth, another important limitation is that the CIA model used in this study is not a disease-specific model of AOSD. Although CIA shares several inflammatory characteristics with AOSD, the observed associations primarily support the inflammatory activity of the identified microbial taxa rather than directly validating AOSD-specific mechanisms. Therefore, caution should be exercised when extrapolating these findings to human AOSD. Finally, although MR strengthens potential genetically predicted associations, its results inherently depend on genetic instruments and underlying assumptions; thus, further validation through large-scale prospective cohorts and experimental functional studies is warranted.

## 5 Conclusion

In conclusion, this study integrates genetic epidemiological analyses with experimental support to investigate the potential associations between gut microbiota and AOSD susceptibility. Using MR and immune mediation analyses, several microbial taxa, including *P. goldsteinii*, *A. shahii*, and *Streptococcus*, showed potential genetically predicted associations with increased risks of AOSD, whereas *Akkermansia*, and members of the *Verrucomicrobia* lineage were associated with reduced risks*.* These associations are partially mediated by T cell-related immune subsets, highlighting a potential microbiota-immune axis underlying AOSD susceptibility. The CIA experiments showed the pro-inflammatory associations of *P. goldsteinii* and *A. shahii*, and provided biological support for the inflammatory plausibility of the MR findings. However, because CIA is not a disease-specific model of AOSD, these findings should not be interpreted as direct validation of AOSD pathogenesis, and further mechanistic studies are warranted. Given the limited number of AOSD cases and the substantial multiple-testing burden, these findings should be interpreted cautiously and warrant validation in larger independent cohorts and mechanistic studies. Overall, these findings suggest that gut microbiotas may be associated with AOSD susceptibility and immune dysregulation, and they provide a foundation for future mechanistic studies and microbiome-based therapeutic strategies.

## Data Availability

The original contributions presented in the study are included in the article/Supplementary Material, further inquiries can be directed to the corresponding author.
