# Supplementary material for: Deciphering the microbiota-immune axis in adult-onset Still’s disease: evidence from Mendelian randomization and experimental evaluation
Source: Front Genet. 2026 Oct 19;17:1841584. doi: 10.3389/fgene.2026.1841584 (PMC13724513; doi:10.3389/fgene.2026.1841584)
Supplement: Supplementary file 1 [file DataSheet1.docx]

**STROBE-MR Checklist of items to address in reports of Mendelian randomization studies**^1^ ^2^

|  | **CORRESPONDING AUTHOR:** |  |  |
| --- | --- | --- | --- |
|  | **MANUSCRIPT TITLE:** |  |  |
| **Item No.** | **Section** | **Checklist item** | **Please provide summary information or confirmation (*where indicated*) to enable assessment of your manuscript without referring to the manuscript. Also, include page, section name and paragraph. Note that some items require only confirmation and page/section details.** |
| 1 | **TITLE and ABSTRACT** | Include Mendelian randomization (MR) in the title and abstract ***(only confirm that MR has been included)****.* | Yes, we have included the Mendelian randomization in the title and abstract. |
|  | **INTRODUCTION** |  |  |
| 2 | **Background** | Explain the scientific background and rationale for the study. What is the exposure? Is a potential causal relationship between exposure and outcome plausible? Justify why MR is a helpful method to address the study question. | **Scientific Background:**  Adult-onset Still’s disease (AOSD) is a rare systemic autoinflammatory disorder characterized by excessive and dysregulated inflammatory responses, typically manifesting as high fever, skin rash, systemic inflammation, and polyarthritis or diffuse arthritis. Unlike vulgaris arthritis, AOSD is associated with substantial morbidity and mortality, particularly in patients complicated by macrophage activation syndrome (MAS), a life-threatening hyperinflammatory syndrome driven by excessive immune activation. The exact etiologies of AOSD remain unclear, but current evidence suggests that the disease involves abnormal immune activation driven by genetic predispositions and environmental factors. Genome-wide association studies (GWAS) have identified several polymorphisms in human leukocyte antigen (HLA) genes, suggesting a genetic basis for the disease. Additionally, there is emerging evidence that gut microbiota, which regulate immune responses in several autoimmune disorders, may play a role in AOSD. Gut microbes help maintain immune homeostasis and regulate immune function, as observed in diseases like rheumatoid arthritis, Sjogren’s syndrome, and systemic lupus erythematosus. This suggests that gut microbial dysbiosis could contribute to abnormal immune responses and potentially drive AOSD's pathogenesis. However, the genetical relationship between gut microbiota dysfunction and the risk of AOSD has yet to be fully established.  **Exposure:** The exposure of interest in this study is 412 Gut microbiota.  **Potential Causal Relationship:** The hypothesis that microbial dysbiosis drives abnormal immune responses, leading to AOSD, is biologically plausible given the role of the gut microbiota in modulating immune function in autoimmune disorders. Evidence from other diseases, such as RA, Sjogren’s syndrome, and SLE, supports the idea that gut microbiota can influence the onset and progression of systemic autoimmune diseases. Thus, a potential genetically predicted association between gut microbiota dysfunction and AOSD is plausible, but it has not been definitively established.  **Justification for Mendelian Randomization (MR):** Mendelian randomization (MR) is a powerful genetic epidemiological approach that uses genetic variants as instrumental variables (IVs) to infer potential genetically predicted associations between exposures and disease outcomes. Compared with conventional observational studies, MR analysis significantly reduces biases arising from residual confounding and reverse interference, thereby providing more robust evidence for potential genetically predicted associations. In parallel, accumulating evidences have highlighted the central role of immune dysregulation in AOSD susceptibility, involving multiple immune-cell subsets and cytokine signaling pathways, particularly those mediated by interleukin-1/6 (IL-1/6) and interferons (IFNs). By comprehensively integrating 731 immune-cell traits, we can identify specific immune-cell subsets and immune-related traits as potential mediators between gut microbial dysbiosis and the onset or development of AOSD. |
| 3 | **Objectives** | State specific objectives clearly, including pre-specified causal hypotheses (if any). | **Objectives:**   a) To investigate genetically predicted associations between 412 gut microbiota taxa and AOSD susceptibility using bidirectional two-sample MR analysis. b) To investigate genetically predicted associations between 731 immune cell traits and AOSD susceptibility using bidirectional two-sample MR analysis.   c) To evaluate whether immune cell traits may mediate the associations between gut microbiota and AOSD using a two-step MR framework. |
|  | **METHODS** |  |  |
| 4 | **Study design and data sources** | Present key elements of the study design. Include a table listing sources of data for all phases of the study, if required.  For each data source contributing to the analysis, describe the following: |  |
|  | a) | **Setting**: Describe the study design and the underlying population. Describe the setting, locations, and relevant dates, including periods of recruitment, exposure, follow-up, and data collection, when available. ***Only confirm that this is in the manuscript and provide page/section.*** | In this study, a two-sample MR approach was applied to investigate the potential associations between gut microbiota and the risks of AOSD. To further understand the mediation induced by immune-cell traits, we employed a two-step MR strategy to calculate mediation effects and validated the proinflammatory effects of critical microbiota on AOSD in vivo. The study's design and analytical workflow are summarized in Figure 1. |
|  | b) | **Participants**: Give the eligibility criteria, and the sources and methods of selection of participants. Report the sample size, and whether any power or sample size calculations were carried out prior to the main analysis. ***Only confirm that this is in the manuscript and provide page/section.*** | GWAS summary statistic datasets of Exposure, Mediator, and Outcomes were summarized in the Method section. |
|  | c) | Describe measurement, quality control and selection of genetic variants. | We have described measurement, quality control and selection of genetic variants in Method section. |
|  | d) | For each exposure, outcome, and other relevant variables, describe methods of assessment and diagnostic criteria for diseases. | We have described the methods of assessment and diagnostic criteria for diseases for each exposure, outcome, and other relevant variables. |
|  | e) | Provide details of ethics committee approval and participant informed consent. ***Only confirm that this is in the manuscript and provide page/section.*** | The Ethics statement is listed in the additional section after Discussion. |
| 5 | **Assumptions** | Clearly state the three core IV assumptions for the main analysis (relevance, independence and exclusion restriction) as well as assumptions for any additional or sensitivity analysis. ***Only confirm that this is in the manuscript and provide page/section.*** | Yes, the core IV assumptions for the main analysis hve been stated in “MR analysis and Sensitivity analysis” parts in Methods section. |
| 6 | **Statistical methods: main analysis** | Describe statistical methods and statistics used. |  |
|  | a) | Describe how quantitative variables were handled in the analyses (i.e., scale, units, model). | Our analysis is based on the SNPs of GWAS summary datasets, there is no quantitative variables for this study. |
|  | b) | Describe how genetic variants were handled in the analyses and, if applicable, how their weights were selected. | Genetic instruments were selected based on potentially genetic associations (P < 1e-5) and linkage disequilibrium clumping criteria (r² < 0.01 within a 10,000 kb window). Potentially pleiotropic or outcome-associated instrumental variables were excluded based on their associations with the AOSD outcome (P < 0.01), to minimize potential bias from horizontal pleiotropy and reverse genetical effects. |
|  | c) | Describe the MR estimator (e.g., two-stage least squares, Wald ratio) and related statistics. Provide details of the included covariates and, in case of two-sample MR, whether the same covariate set was used for adjustment in the two samples. | The primary evaluation of MR results was conducted using the inverse-variance weighted (IVW) method, complemented with estimations of the MR‐Egger and weighted median (WM) methods using the TwoSampleMR package. |
|  | d) | Explain how missing data were addressed. ***Only confirm that this is in the manuscript and provide page/section.*** | The GWAS datasets for each variable and there is no missing data for this MR study. |
|  | e) | Explain how multiple testing was addressed, if applicable. ***Only confirm that this is in the manuscript and provide page/section.*** | To account for the substantial multiple-testing burden across different analytical stages, false discovery rate (FDR) correction with Benjamini-Hochberg (BH) methods was applied separately within each analysis category. |
| 7 | **Assessment of assumptions** | Describe any methods or prior knowledge used to assess the assumptions or justify their validity. | Potentially pleiotropic IVs were identified using the PhenoScanner database and excluded if they were associated with known confounders or the AOSD outcome (P <0.01). The primary evaluation of MR results was conducted using the inverse-variance weighted (IVW) method, complemented with estimations from MR‐Egger, weighted median (WM), Simple mode, and Weighted mode methods, using the TwoSampleMR package (https://github.com/MRCIEU/TwoSampleMR) |
| 8 | **Sensitivity analyses and additional analyses** | Describe any sensitivity analyses or additional analyses performed (e.g., comparison of effect estimates from different approaches, independent replication, bias analytic techniques, validation of instruments, simulations). | For sensitivity analysis, heterogeneity among the instrumental estimates was assessed using Cochran’s Q test, with P >0.05 indicating no statistically significant heterogeneity. Horizontal pleiotropy was evaluated using the intercept from MR-Egger regression and the global test of the MR‐pleiotropy residual sum and outlier (MR-PRESSO) test, with P >0.05 indicating no evidence of statistically significant horizontal pleiotropy. To further enhance the robustness of our findings, the analyses were independently repeated using the Bayesian weighted Mendelian randomization (BWMR) method, and only microbiota with significance after BH correction (BH-adjusted p-values <0.05) in both IVW and BWMR analyses were identified as having potential genetically predicted associations with AOSD susceptibility. |
| 9 | **Software and pre-registration** |  |  |
|  | a) | Provide details of statistical software and package(s), including version and settings used. ***Only confirm that this is in the manuscript and provide page/section.*** | All statistical analyses were performed using R software (version 4.3.2) and corresponding R package has been listed in methods sections. |
|  | b) | State whether the study protocol and details were pre-registered (as well as when and where). | All GWAS summary statistics used in this study were derived from previously published studies, which received approval from corresponding ethical committees and obtained the informed consent forms from all participants. |
|  | **RESULTS** |  |  |
| 10 | **Descriptive data** |  |  |
|  | a) | Report the numbers of individuals at each stage of studies included and reasons for exclusion. Consider the use of a flow diagram. | Comprehensive details of all individuals were provided in **Supplementary Table 1.** |
|  | b) | Report summary statistics for phenotypic exposure(s), outcome(s), and other relevant variables (e.g., means, SDs, proportions). | Utilizing the IVW and BWMR methods, 10 bacterial taxa and 3 related metabolic pathways exhibited significant potential genetically predicted associations with AOSD traits |
|  | c) | If the data sources include meta-analyses of previous studies, provide the assessments of heterogeneity across these studies. ***Only confirm that this is in the manuscript and provide page/section.*** | There is no meta-analyses of previous studies in this study. |
|  | d) | For two-sample MR:  i.  Provide justification of the similarity of the genetic variant-exposure associations between the exposure and outcome samples.  ii.  Provide information on the number of individuals who overlap between the exposure and outcome studies. | After BH correction for 412 microbiota-related exposures, 13 associations remained statistically significant (BH-adjusted P< 0.05), including 10 microbial taxa and 3 microbial metabolic pathways. All findings reported below refer to associations that remained significant after multiple-testing correction unless otherwise stated. (Figure. 2). These findings included significant potential associations of Family Streptococcaceae (OR: 2.66; 95% CI: 1.37-5.17, BH-adjusted P=0.019), Species Alistipes shahii (OR: 3.22; 95% CI: 1.03-10.10, BH-adjusted P=0.045), Genus Streptococcus (OR: 3.48; 95% CI: 1.52-7.99, BH-adjusted P=0.019), Species Parabacteroides distasonis (OR: 3.94; 95% CI: 1.39-11.21, BH-adjusted P=0.028), Species Parabacteroides goldsteinii (OR: 2.34; 95% CI: 1.28-4.28, BH-adjusted P=0.019), Creatinine degradation (OR: 4.05; 95% CI: 1.53-10.72, BH-adjusted P=0.019), and L-tryptophan biosynthesis (OR: 3.62; 95% CI: 1.13-11.58, BH-adjusted P=0.043) with increased risks in AOSD outcomes. |
| 11 | **Main results** |  |  |
|  | a) | Report the associations between genetic variant and exposure, and between genetic variant and outcome, preferably on an interpretable scale. | In contrast, several taxa and pathways displayed significant protective roles in the genetic predisposition to AOSD, including Verrucomicrobiae at Class, Family, Genus and Phylum levels (OR: 0.33; 95% CI: 0.12-0.92, BH-adjusted P=0.043), Genus Akkermansia (OR: 0.33; 95% CI: 0.12-0.92, BH-adjusted P=0.045), and Mannan degradation (OR: 0.28; 95% CI: 0.08-0.96, BH-adjusted P=0.048). |
|  | b) | Report MR estimates of the relationship between exposure and outcome, and the measures of uncertainty from the MR analysis, on an interpretable scale, such as odds ratio or relative risk per SD difference. | Among the 731 immune-cell traits evaluated, 28 showed nominal significance in the IVW analysis (nominal P<0.05). Following BH correction, 20 immune-cell traits remained statistically significant (BH-adjusted P<0.05) and were subsequently retained for mediator screening. Multiple immune cells exhibited potential risk for AOSD, including CD25 on CD39+CD4+T cell (OR: 1.31; 95% CI: 1.03-1.65, BH-adjusted P=0.043), CD25hi CD45RA+ CD4 not Treg AC (OR: 1.38; 95% CI: 1.10-1.72, BH-adjusted P=0.028), CD45 on basophil (OR: 1.54; 95% CI: 1.10-2.15, BH-adjusted P=0.035), CD8 on NKT (OR: 1.59; 95% CI: 1.03-2.47, BH-adjusted P=0.048), IgD-CD38br %B cell (OR: 1.85; 95% CI: 1.13-3.04, BH-adjusted P=0.035), and Transitional AC (OR: 1.48; 95% CI: 1.02-2.80, BH-adjusted P=0.043). |
|  | c) | If relevant, translate estimates of relative risk into absolute risk for a meaningful time period. | Conversely, the other immune cells displayed significant protective roles in AOSD, particularly in T-lymphocyte related subsets, such as CD127 on CD4+ T (OR: 0.50; 95% CI: 0.27-0.92, BH-adjusted P=0.043), CD28+ DN (CD4-CD8-) %T cell (OR: 0.66; 95% CI: 0.45-0.98, BH-adjusted P=0.043), CD4 on EM CD4+T (OR: 0.63; 95% CI: 0.47-0.86, BH-adjusted P=0.024), and so on (Figure. 3) |
|  | d) | Plots to visualize results (e.g., forest plot, scatterplot of associations between genetic variants and outcome versus between genetic variants and exposure). | We have applied the forest plot and scatterplot to visualize the MR results |
| 12 | **Assessment of assumptions** |  |  |
|  | a) | Report the assessment of the validity of the assumptions. ***Only confirm that this is in the manuscript and provide page/section.*** | The core instrumental variable assumptions were evaluated through bidirectional MR analysis and sensitivity analyses. Potentially pleiotropic or outcome-associated variants were excluded, and additional assessments, including Cochran’s Q test, MR-Egger intercept analysis, and MR-PRESSO testing, were performed to evaluate heterogeneity and horizontal pleiotropy. |
|  | b) | Report any additional statistics (e.g., assessments of heterogeneity across genetic variants, such as *I^2^*, Q statistic or E-value). ***Only confirm that this is in the manuscript and provide page/section.*** | For sensitivity analysis, heterogeneity among the instrumental estimates was assessed using Cochran’s Q test, with P >0.05 indicating no statistically significant heterogeneity, and examined the average horizontal pleiotropy using the intercept from MR‐Egger regression and MR-pleiotropy residual sum and outlier (MR-PRESSO) test P >0.05 indicating no evidence of statistically significant horizontal pleiotropy. |
| 13 | **Sensitivity analyses and additional analyses** |  |  |
|  | a) | Report any sensitivity analyses to assess the robustness of the main results to violations of the assumptions. | Notably, the sensitivity analysis indicated that heterogeneity existed in Genus Akkermansia and Verrucomicrobia at Class, Family, Genus and Phylum levels, but no pleiotropy existing in all 13 taxa and pathways |
|  | b) | Report results from other sensitivity analyses or additional analyses. | Similarly, no heterogeneity and pleiotropy were found in these immune cells (**Supplementary Table 3**). |
|  | c) | Report any assessment of direction of causal relationship (e.g., bidirectional MR). | For significant gut microorganisms, reverse MR analysis demonstrated the absence of reverse risk associations for all 13 taxa and pathways. |
|  | d) | When relevant, report and compare with estimates from non-MR analyses. | There is no significant relevant relationship for significant taxa in reverse MR analysis. |
|  | e) | Additional plots to visualize results (e.g., leave-one-out analyses). | The results of reverse MR are displayed in Table 1. |
|  | **DISCUSSION** |  |  |
| 14 | **Key results** | Summarize key results with reference to study objectives. | Our study explored the potential genetically predicted associations between specific microbiota taxa and AOSD through MR analysis, providing promising insights into the microbiota-immune axis underlying AOSD. The Verrucomicrobia (phylum), Verrucomicrobiae (class), Verrucomicrobiales (order), Verrucomicrobiaceae (family), and Akkermansia (genus) were inversely associated with AOSD risk, suggesting that genetically predicted higher abundance of these taxa may be associated with a lower susceptibility to AOSD. Conversely, the Streptococcaceae (family), Streptococcus (genus), Alistipes shahii (species), Parabacteroides goldsteinii (species), and Parabacteroides distasonis (species) were positively associated with AOSD risk, indicating that these taxa might have potential associations with increased susceptibility to the disease. |
| 15 | **Limitations** | Discuss limitations of the study, taking into account the validity of the IV assumptions, other sources of potential bias, and imprecision. Discuss both direction and magnitude of any potential bias and any efforts to address them. | However, there are still several limitations in our study. Firstly, the study populations were restricted to individuals of European ancestry, which may limit the generalizability of the findings to other ethnic groups. Secondly, the use of GWAS summary-level data precluded access to individual-level clinical variables, including age, sex, and disease severity, thereby limiting more refined stratified or phenotypic analyses. Third, the FinnGen’s GWAS dataset of AOSD included only 113 cases, reflecting the rarity of AOSD but also representing a potential limitation for MR analysis. The relatively small sample size may reduce statistical power and increase the risk of false-negative findings or unstable effect estimates, particularly for associations with modest effect sizes. Given the limited number of AOSD cases, our findings should still be interpreted cautiously and require further validation in larger independent cohorts and multicenter studies. Moreover, another important limitation is that the CIA model used in this study is not a disease-specific model of AOSD. Although CIA shares several inflammatory characteristics with AOSD, the observed associations primarily support the inflammatory activity of the identified microbial taxa rather than directly validating AOSD-specific mechanisms. Therefore, caution should be exercised when extrapolating these findings to human AOSD. Finally, although MR strengthens potential genetically predicted associations, the results remain dependent on genetic instruments and assumptions; thus, further validation through large-scale prospective cohorts and experimental functional studies is warranted. |
| 16 | **Interpretation** |  |  |
|  | a) | **Meaning**: Give a cautious overall interpretation of results in the context of their limitations and in comparison with other studies. | In conclusion, this study integrates genetic epidemiology and experimental evidence to investigate the potential association between gut microbiota and AOSD susceptibility. |
|  | b) | **Mechanism**: Discuss underlying biological mechanisms that could drive a potential causal relationship between the investigated exposure and the outcome, and whether the gene-environment equivalence assumption is reasonable. Use causal language carefully, clarifying that IV estimates may provide causal effects only under certain assumptions. | Using MR and immune mediation analyses, several microbial taxa, including Parabacteroides goldsteinii, Alistipes shahii, and Streptococcus, showed potential genetically predicted associations with increased risks of AOSD, whereas Akkermansia, and members of the Verrucomicrobia lineage were associated with reduced risks. These associations are partially mediated by T cell-related immune subsets, highlighting a potential microbiota-immune axis underlying AOSD susceptibility. The CIA experiments showed the pro-inflammatory associations of P. goldsteinii and A. shahii, and provided biological support for the inflammatory plausibility of the MR findings. However, because CIA is not a disease-specific model of AOSD, these findings should not be interpreted as direct validation of AOSD pathogenesis, and further mechanistic studies are warranted. Given the limited number of AOSD cases and the substantial multiple-testing burden, these findings should be interpreted cautiously and warrant validation in larger independent cohorts and mechanistic studies. Overall, these findings suggest that gut microbiotas may be associated with AOSD’s susceptibility and immune dysregulation, and provide a foundation for future mechanistic studies and microbiome-based therapeutic strategies. |
|  | c) | **Clinical relevance**: Discuss whether the results have clinical or public policy relevance, and to what extent they inform effect sizes of possible interventions. | There is no clinical relevance for MR analysis |
| 17 | **Generalizability** | Discuss the generalizability of the study results (a) to other populations, (b) across other exposure periods/timings, and (c) across other levels of exposure. | The study population was restricted to individuals of European ancestry, which may limit the generalizability of the findings to other ethnic groups. |
|  | **OTHER INFORMATION** |  |  |
| 18 | **Data and data sharing** | Provide the data used to perform all analyses or report where and how the data can be accessed, and reference these sources in the article. Provide the statistical code needed to reproduce the results in the article, or report whether the code is publicly accessible and if so, where this can be found. | Datasets used in our study are available from the corresponding studies and summarized in **Supplementary Table 1**. The GWAS summary statistics are publicly downloaded according to Supplementary material. The statistical codes are available upon reasonable request. |

This checklist is adapted from the “[STROBE-MR checklist](https://www.strobe-mr.org/)” by the Equator Network, used under the Creative Commons Attribution 3.0 Unported (CC BY 3.0) license.

1.	Skrivankova VW, Richmond RC, Woolf BAR, Yarmolinsky J, Davies NM, Swanson SA, et al. Strengthening the Reporting of Observational Studies in Epidemiology Using Mendelian Randomization: The STROBE-MR Statement. JAMA. 2021; Oct 26;326(16):1614-1621. doi: 10.1001/jama.2021.18236.

2.	Skrivankova VW, Richmond RC, Woolf BAR, Davies NM, Swanson SA, VanderWeele TJ, et al. Strengthening the Reporting of Observational Studies in Epidemiology using Mendelian Randomisation (STROBE-MR): Explanation and Elaboration. BMJ. 2021;375:n2233. doi: 10.1136/bmj.n2233.

**Supplementary Tables:**

**Supplementary Table 1. Source information of GWAS summary datasets for Exposure, Mediator, and Outcome.**

| **Type of dataset** | **Data subtype** | **Resource** | **Sample size** | **Population** | **Reference** |
| --- | --- | --- | --- | --- | --- |
| Exposure | 207 bacterial taxa and 205 microbiota-associated pathways | Dutch Microbiome Project | 7,738 | European | [Esteban A, et al. Nat Genet. 2022. PMID: 35115690. Effect of host genetics on the gut microbiome in 7,738 participants of the Dutch Microbiome Project.](https://www.nature.com/articles/s41588-021-00913-z) |
| Mediator | 731 immune cell traits | Orrù V, et al. study | 3,757 | European | [Orrù V, et al. Nat Genet. 2020. PMID: 32929287. Complex genetic signatures in immune cells underlie autoimmunity and inform therapy.](https://www.nature.com/articles/s41588-021-00913-z) |
| Outcome | Adult-onset Still disease (AOSD) | FinnGen: STILL_ADULT | case:113, control:341,455 | European | gs://finngen-public-data-r10/summary_stats/finngen_R10_STILL_ADULT.gz |

**Supplementary Table 2.** **Results of MR and Sensitivity analysis on the association between gut microbiota and AOSD outcomes.**

| **Gut microbiota and pathways** | **MR method** | **No. of SNP** | **OR (95%CI)** | ***P*-value** | **Heterogeneity test** | | **Pleiotropy test** | |
| --- | --- | --- | --- | --- | --- | --- | --- | --- |
|  |  |  |  |  | **Cochran's Q** | ***P*** | **intercept** | ***P*** |
| **Creatinine degradation** | MR Egger | 9 | 11.79 (0.58-240.04) | 0.152685737 | 10.587 | 0.158 | -0.191 | 0.456 |
|  | Weighted median | 9 | 5.28 (1.87-14.93) | 0.001705771 | NA | NA | NA | NA |
|  | Inverse variance weighted | 9 | 3.72 (1.49-9.29) | 0.004841468 | 11.528 | 0.174 | NA | NA |
|  | Simple mode | 9 | 8.37 (1.40-49.86) | 0.047989076 | NA | NA | NA | NA |
|  | Weighted mode | 9 | 6.70 (1.47-30.51) | 0.039456328 | NA | NA | NA | NA |
|  | MR-PRESSO | NA | NA | 0.246 | NA | NA | NA | NA |
|  |  |  |  |  |  |  |  |  |
| **Glycogen biosynthesis from ADP D Glucose** | MR Egger | 11 | 0.04 (0.001-1.31) | 0.103792987 | 5.518 | 0.787 | 0.216 | 0.239 |
|  | Weighted median | 11 | 0.33 (0.08-1.28) | 0.10738031 | NA | NA | NA | NA |
|  | Inverse variance weighted | 11 | 0.34 (0.12-0.94) | 0.03787814 | 7.107 | 0.715 | NA | NA |
|  | Simple mode | 11 | 0.31 (0.04-2.56) | 0.30399732 | NA | NA | NA | NA |
|  | Weighted mode | 11 | 0.29 (0.05-1.83) | 0.216434451 | NA | NA | NA | NA |
|  | MR-PRESSO | NA | NA | 0.751 | NA | NA | NA | NA |
|  |  |  |  |  |  |  |  |  |
| **CMP 3 deoxy D manno octulosonate biosynthesis** | MR Egger | 6 | 47.43 (0.02-12063) | 0.389357035 | 2.532 | 0.639 | -0.586 | 0.169 |
|  | Weighted median | 6 | 0.03 (0.002-0.25) | 0.001649378 | NA | NA | NA | NA |
|  | Inverse variance weighted | 6 | 0.07 (0.01-0.33) | 0.000865057 | 5.340 | 0.376 | NA | NA |
|  | Simple mode | 6 | 0.02 (0.001-0.61) | 0.075481977 | NA | NA | NA | NA |
|  | Weighted mode | 6 | 0.02 (0.001-0.70) | 0.084224058 | NA | NA | NA | NA |
|  | MR-PRESSO | NA | NA | 0.427 | NA | NA | NA | NA |
|  |  |  |  |  |  |  |  |  |
| **Mannan degradation** | MR Egger | 7 | 0.18 (0.001-53.07) | 0.578172629 | 2.379 | 0.795 | 0.064 | 0.871 |
|  | Weighted median | 7 | 0.21 (0.05-0.99) | 0.049024414 | NA | NA | NA | NA |
|  | Inverse variance weighted | 7 | 0.29 (0.09-0.92) | 0.035286419 | 2.408 | 0.879 | NA | NA |
|  | Simple mode | 7 | 0.16 (0.02-1.50) | 0.160065477 | NA | NA | NA | NA |
|  | Weighted mode | 7 | 0.18 (0.25-1.29) | 0.139167535 | NA | NA | NA | NA |
|  | MR-PRESSO | NA | NA | 0.889 | NA | NA | NA | NA |
|  |  |  |  |  |  |  |  |  |
| **L-tryptophan biosynthesis** | MR Egger | 12 | 0.29 (0.002-40.34) | 0.636072325 | 11.563 | 0.315 | 0.231 | 0.338 |
|  | Weighted median | 12 | 1.69 (0.39-7.29) | 0.48445367 | NA | NA | NA | NA |
|  | Inverse variance weighted | 12 | 3.43 (1.12-10.52) | 0.030913625 | 12.732 | 0.311 | NA | NA |
|  | Simple mode | 12 | 1.22 (0.09-16.27) | 0.881733478 | NA | NA | NA | NA |
|  | Weighted mode | 12 | 1.25 (0.09-17.89) | 0.87285809 | NA | NA | NA | NA |
|  | MR-PRESSO | NA | NA | 0.349 | NA | NA | NA | NA |
|  |  |  |  |  |  |  |  |  |
| **Class Verrucomicrobiae*** | MR Egger | 15 | 0.56 (0.004-85.45) | 0.824740528 | 23.351 | 0.038 | -0.055 | 0.841 |
|  | Weighted median | 15 | 0.42 (0.14-1.27) | 0.126447917 | NA | NA | NA | NA |
|  | Inverse variance weighted | 15 | 0.33 (0.13-0.88) | 0.027127469 | 2.43 | 0.054 | NA | NA |
|  | Simple mode | 15 | 0.58 (0.10-3.41) | 0.558818702 | NA | NA | NA | NA |
|  | Weighted mode | 15 | 0.45 (0.08-2.42) | 0.365285404 | NA | NA | NA | NA |
|  | MR-PRESSO | NA | NA | 0.058 | NA | NA | NA | NA |
|  |  |  |  |  |  |  |  |  |
| **Family Streptococcaceae** | MR Egger | 14 | 0.58 (0.04-9.16) | 0.708115466 | 11.00 | 0.528 | 0.223 | 0.306 |
|  | Weighted median | 14 | 2.79 (1.17-6.64) | 0.020125482 | NA | NA | NA | NA |
|  | Inverse variance weighted | 14 | 2.52 (1.36-4.67) | 0.003341284 | 12.15 | 0.516 | NA | NA |
|  | Simple mode | 14 | 3.09 (0.64-14.82) | 0.182644728 | NA | NA | NA | NA |
|  | Weighted mode | 14 | 3.12 (0.74-13.24) | 0.14553091 | NA | NA | NA | NA |
|  | MR-PRESSO | NA | NA | 0.546 | NA | NA | NA | NA |
|  |  |  |  |  |  |  |  |  |
| **Family Verrucomicrobiaceae*** | MR Egger | 15 | 0.56 (0.004-85.27) | 0.824265648 | 23.25 | 0.038 | -0.055 | 0.270 |
|  | Weighted median | 15 | 0.42 (0.14-1.25) | 0.119280337 | NA | NA | NA | NA |
|  | Inverse variance weighted | 15 | 0.33 (0.13-0.88) | 0.027115675 | 23.43 | 0.054 | NA | NA |
|  | Simple mode | 15 | 0.58 (0.09-3.99) | 0.591343255 | NA | NA | NA | NA |
|  | Weighted mode | 15 | 0.45 (0.09-2.34) | 0.35652848 | NA | NA | NA | NA |
|  | MR-PRESSO | NA | NA | 0.065 | NA | NA | NA | NA |
|  |  |  |  |  |  |  |  |  |
| **Genus Streptococcus** | MR Egger | 8 | 3.09 (0.29-324.47) | 0.651097074 | 3.208 | 0.783 | 0.006 | 0.986 |
|  | Weighted median | 8 | 2.82 (0.96-8.33) | 0.060326477 | NA | NA | NA | NA |
|  | Inverse variance weighted | 8 | 3.22 (1.39-7.47) | 0.006435858 | 3.209 | 0.865 | NA | NA |
|  | Simple mode | 8 | 2.09 (0.39-11.18) | 0.416863213 | NA | NA | NA | NA |
|  | Weighted mode | 8 | 2.11 (0.43-10.31) | 0.385921277 | NA | NA | NA | NA |
|  | MR-PRESSO | NA | NA | 0.888 | NA | NA | NA | NA |
|  |  |  |  |  |  |  |  |  |
| **Genus Akkermansia*** | MR Egger | 15 | 0.56 (0.004-85.65) | 0.822839715 | 23.349 | 0.037 | -0.054 | 0.843 |
|  | Weighted median | 15 | 0.42 (0.14-1.24) | 0.118183814 | NA | NA | NA | NA |
|  | Inverse variance weighted | 15 | 0.33 (0.12-0.88) | 0.027070918 | 23.422 | 0.053 | NA | NA |
|  | Simple mode | 15 | 0.58 (0.08-4.13) | 0.598353979 | NA | NA | NA | NA |
|  | Weighted mode | 15 | 0.45 (0.08-2.49) | 0.374268812 | NA | NA | NA | NA |
|  | MR-PRESSO | NA | NA | 0.070 | NA | NA | NA | NA |
|  |  |  |  |  |  |  |  |  |
| **Order Verrucomicrobiales*** | MR Egger | 15 | 0.56 (0.004-85.37) | 0.824479058 | 23.351 | 0.038 | -0.055 | 0.841 |
|  | Weighted median | 15 | 0.42 (0.14-1.28) | 0.129375913 | NA | NA | NA | NA |
|  | Inverse variance weighted | 15 | 0.33 (0.12-0.88) | 0.027120859 | 23.426 | 0.054 | NA | NA |
|  | Simple mode | 15 | 0.58 (0.08-4.03) | 0.592689418 | NA | NA | NA | NA |
|  | Weighted mode | 15 | 0.45 (0.09-2.29) | 0.350561379 | NA | NA | NA | NA |
|  | MR-PRESSO | NA | NA | 0.67 | NA | NA | NA | NA |
|  |  |  |  |  |  |  |  |  |
| **Phylum Verrucomicrobia*** | MR Egger | 15 | 0.56 (0.004-85.47) | 0.825046212 | 23.351 | 0.038 | -0.055 | 0.840 |
|  | Weighted median | 15 | 0.42 (0.14-1.31) | 0.135307915 | NA | NA | NA | NA |
|  | Inverse variance weighted | 15 | 0.33 (0.13-0.88) | 0.027135907 | 23.427 | 0.054 | NA | NA |
|  | Simple mode | 15 | 0.58 (0.09-3.81) | 0.581685177 | NA | NA | NA | NA |
|  | Weighted mode | 15 | 0.45 (0.08-2.46) | 0.369390841 | NA | NA | NA | NA |
|  | MR-PRESSO | NA | NA | 0.070 | NA | NA | NA | NA |
|  |  |  |  |  |  |  |  |  |
| **Species Parabacteroides_distasonis** | MR Egger | 8 | 14.30 (0.44-464.27) | 0.184714482 | 6.187 | 0.403 | -0.146 | 0.453 |
|  | Weighted median | 8 | 4.29 (1.04-17.76) | 0.044401266 | NA | NA | NA | NA |
|  | Inverse variance weighted | 8 | 3.67 (1.31-10.33) | 0.013585034 | 6.852 | 0.444 | NA | NA |
|  | Simple mode | 8 | 7.33 (0.93-57.64) | 0.100102976 | NA | NA | NA | NA |
|  | Weighted mode | 8 | 6.82 (0.87-53.26) | 0.109892263 | NA | NA | NA | NA |
|  | MR-PRESSO | NA | NA | 0.474 | NA | NA | NA | NA |
|  |  |  |  |  |  |  |  |  |
| **Species Parabacteroides_goldsteinii** | MR Egger | 7 | 5.59 (0.11-284.74) | 0.429954 | 0.653 | 0.985 | -0.175 | 0.677 |
|  | Weighted median | 7 | 2.43 (1.21-4.88) | 0.012165 | NA | NA | NA | NA |
|  | Inverse variance weighted | 7 | 2.32 (1.32-4.09) | 0.003533 | 0.849 | 0.991 | NA | NA |
|  | Simple mode | 7 | 2.39 (0.86-6.62) | 0.145278 | NA | NA | NA | NA |
|  | Weighted mode | 7 | 2.38 (0.88-6.46) | 0.139065 | NA | NA | NA | NA |
|  | MR-PRESSO | NA | NA | 0.993 | NA | NA | NA | NA |
|  |  |  |  |  |  |  |  |  |
| **Species Alistipes_shahii** | MR Egger | 11 | 2.87 (0.01-698.94) | 0.716111248 | 6.728 | 0.665 | 0.009 | 0.976 |
|  | Weighted median | 11 | 3.64 (0.87-15.23) | 0.076555694 | NA | NA | NA | NA |
|  | Inverse variance weighted | 11 | 3.12 (1.06-9.16) | 0.038747352 | 6.729 | 0.751 | NA | NA |
|  | Simple mode | 11 | 3.91 (0.39-39.54) | 0.274371042 | NA | NA | NA | NA |
|  | Weighted mode | 11 | 3.48 (0.36-33.94) | 0.307992899 | NA | NA | NA | NA |
|  | MR-PRESSO | NA | NA | 0.768 | NA | NA | NA | NA |

Note: NA, not applicable. Cochran's Q test (Q-P value >0.05) indicates no heterogeneity exists. The intercept of MR Egger is close to 0 with P >0.05 and MR-PRESSO’s Global test > 0·05 indicates no horizontal pleiotropic outliers.

**Supplementary Table 3. Results of MR and Sensitivity analysis on the association between immune cells and AOSD outcomes.**

| **Immune cells** | **MR method** | **No. of SNP** | **OR (95%CI)** | ***P*-value** | **Heterogeneity test** | | **Pleiotropy test** | |
| --- | --- | --- | --- | --- | --- | --- | --- | --- |
|  |  |  |  |  | **Cochran's Q** | ***P*** | **intercept** | ***P*** |
| **IgD- CD38br %B cell** | MR Egger | 17 | 1.67 (0.58-4.81) | 0.35631805 | 11.552 | 0.713 | 0.022 | 0.833 |
|  | Weighted median | 17 | 1.94 (0.96-3.92) | 0.062980401 | NA | NA | NA | NA |
|  | Inverse variance weighted | 17 | 1.85 (1.12-3.04) | 0.015163518 | 11.599 | 0.771 | NA | NA |
|  | Simple mode | 17 | 1.61 (0.55-4.68) | 0.398982482 | NA | NA | NA | NA |
|  | Weighted mode | 17 | 1.62 (0.58-4.57) | 0.372383001 | NA | NA | NA | NA |
|  | MR-PRESSO | NA | NA | 0.753 | NA | NA | NA | NA |
|  |  |  |  |  |  |  |  |  |
| **CD11c+ monocyte %monocyte** | MR Egger | 20 | 0.75 (0.39-1.45) | 0.406439752 | 15.593 | 0.621 | -0.036 | 0.691 |
|  | Weighted median | 20 | 0.75 (0.43-1.30) | 0.309235279 | NA | NA | NA | NA |
|  | Inverse variance weighted | 20 | 0.67 (0.47-0.97) | 0.03545564 | 15.755 | 0.674 | NA | NA |
|  | Simple mode | 20 | 0.89 (0.40-1.99) | 0.778039401 | NA | NA | NA | NA |
|  | Weighted mode | 20 | 0.82 (0.47-1.42) | 0.487326223 | NA | NA | NA | NA |
|  | MR-PRESSO | NA | NA | 0.723 | NA | NA | NA | NA |
|  |  |  |  |  |  |  |  |  |
| **Plasmacytoid DC AC** | MR Egger | 29 | 0.68 (0.51-0.91) | 0.01499689 | 39.696 | 0.055 | 0.089 | 0.182 |
|  | Weighted median | 29 | 0.83 (0.61-1.13) | 0.232362882 | NA | NA | NA | NA |
|  | Inverse variance weighted | 29 | 0.77 (0.62-0.97) | 0.027176304 | 42.447 | 0.039 | NA | NA |
|  | Simple mode | 29 | 0.74 (0.40-1.35) | 0.331390332 | NA | NA | NA | NA |
|  | Weighted mode | 29 | 0.78 (0.57-1.06) | 0.120337398 | NA | NA | NA | NA |
|  | MR-PRESSO | NA | NA | 0.057 | NA | NA | NA | NA |
|  |  |  |  |  |  |  |  |  |
| **CD62L- CD86+ myeloid DC AC** | MR Egger | 19 | 0.70 (0.48-1.03) | 0.084860906 | 16.152 | 0.513 | -0.019 | 0.811 |
|  | Weighted median | 19 | 0.69 (0.48-1.00) | 0.052221421 | NA | NA | NA | NA |
|  | Inverse variance weighted | 19 | 0.68 (0.52-0.90) | 0.006454234 | 16.211 | 0.578 | NA | NA |
|  | Simple mode | 19 | 0.62 (0.33-1.16) | 0.150930806 | NA | NA | NA | NA |
|  | Weighted mode | 19 | 0.68 (0.49-0.95) | 0.038624925 | NA | NA | NA | NA |
|  | MR-PRESSO | NA | NA | 0.685 | NA | NA | NA | NA |
|  |  |  |  |  |  |  |  |  |
| **CD25hi %CD4+ T** | MR Egger | 25 | 0.89 (0.56-1.43) | 0.644948573 | 19.745 | 0.657 | -0.089 | 0.217 |
|  | Weighted median | 25 | 0.77 (0.50-1.19) | 0.239411534 | NA | NA | NA | NA |
|  | Inverse variance weighted | 25 | 0.71 (0.52-0.98) | 0.038937822 | 21.359 | 0.617 | NA | NA |
|  | Simple mode | 25 | 0.71 (0.34-1.49) | 0.374861526 | NA | NA | NA | NA |
|  | Weighted mode | 25 | 0.77 (0.52-1.14) | 0.200350857 | NA | NA | NA | NA |
|  | MR-PRESSO | NA | NA | 0.678 | NA | NA | NA | NA |
|  |  |  |  |  |  |  |  |  |
| **CD25hi CD45RA+ CD4 not Treg AC** | MR Egger | 25 | 1.31 (0.97-1.76) | 0.093580497 | 18.271 | 0.743 | 0.037 | 0.616 |
|  | Weighted median | 25 | 1.57 (1.13-2.19) | 0.007893758 | NA | NA | NA | NA |
|  | Inverse variance weighted | 25 | 1.38 (1.10-1.72) | 0.004411979 | 18.529 | 0.777 | NA | NA |
|  | Simple mode | 25 | 1.30 (0.70-2.41) | 0.409679092 | NA | NA | NA | NA |
|  | Weighted mode | 25 | 1.42 (1.04-1.95) | 0.039376487 | NA | NA | NA | NA |
|  | MR-PRESSO | NA | NA | 0.768 | NA | NA | NA | NA |
|  |  |  |  |  |  |  |  |  |
| **CD33dim HLA DR+ CD11b- AC** | MR Egger | 27 | 1.19 (0.96-1.47) | 0.134726183 | 18.673 | 0.813 | 0.007 | 0.899 |
|  | Weighted median | 27 | 1.15 (0.88-1.49) | 0.308470998 | NA | NA | NA | NA |
|  | Inverse variance weighted | 27 | 1.20 (1.00-1.43) | 0.045917474 | 18.689 | 0.849 | NA | NA |
|  | Simple mode | 27 | 1.33 (0.84-2.13) | 0.238971328 | NA | NA | NA | NA |
|  | Weighted mode | 27 | 1.19 (0.96-1.49) | 0.125900621 | NA | NA | NA | NA |
|  | MR-PRESSO | NA | NA | 0.856 | NA | NA | NA | NA |
|  |  |  |  |  |  |  |  |  |
| **Transitional AC** | MR Egger | 26 | 1.10 (0.96-1.26) | 0.174151525 | 30.92 | 0.156 | 0.055 | 0.379 |
|  | Weighted median | 26 | 1.11 (0.96-1.28) | 0.169025025 | NA | NA | NA | NA |
|  | Inverse variance weighted | 26 | 1.13 (1.00-1.27) | 0.048042642 | 31.95 | 0.159 | NA | NA |
|  | Simple mode | 26 | 0.94 (0.68-1.30) | 0.712970424 | NA | NA | NA | NA |
|  | Weighted mode | 26 | 1.09 (0.97-1.23) | 0.154036152 | NA | NA | NA | NA |
|  | MR-PRESSO | NA | NA | 0.209 | NA | NA | NA | NA |
|  |  |  |  |  |  |  |  |  |
| **T cell %lymphocyte** | MR Egger | 23 | 0.46 (0.25-0.84) | 0.020741192 | 15.110 | 0.817 | 0.118 | 0.134 |
|  | Weighted median | 23 | 0.55 (0.33-0.92) | 0.02232834 | NA | NA | NA | NA |
|  | Inverse variance weighted | 23 | 0.68 (0.47-0.98) | 0.036498357 | 17.545 | 0.733 | NA | NA |
|  | Simple mode | 23 | 0.53 (0.27-1.07) | 0.089089375 | NA | NA | NA | NA |
|  | Weighted mode | 23 | 0.56 (0.33-0.93) | 0.035938729 | NA | NA | NA | NA |
|  | MR-PRESSO | NA | NA | 0.758 | NA | NA | NA | NA |
|  |  |  |  |  |  |  |  |  |
| **CD28+ DN (CD4-CD8-) %T cell** | MR Egger | 31 | 1.25 (0.70-2.22) | 0.461591977 | 33.155 | 0.272 | 0.047 | 0.446 |
|  | Weighted median | 31 | 1.27 (0.73-2.23) | 0.399960122 | NA | NA | NA | NA |
|  | Inverse variance weighted | 31 | 1.48 (1.02-2.15) | 0.038753876 | 33.839 | 0.287 | NA | NA |
|  | Simple mode | 31 | 1.76 (0.67-4.66) | 0.263002691 | NA | NA | NA | NA |
|  | Weighted mode | 31 | 1.35 (0.82-2.24) | 0.24948546 | NA | NA | NA | NA |
|  | MR-PRESSO | NA | NA | 0.330 | NA | NA | NA | NA |
|  |  |  |  |  |  |  |  |  |
| **CD25++ CD8br %T cell** | MR Egger | 17 | 1.35 (0.27-6.87) | 0.719810103 | 17.993 | 0.263 | -0.195 | 0.237 |
|  | Weighted median | 17 | 0.59 (0.30-1.19) | 0.14071448 | NA | NA | NA | NA |
|  | Inverse variance weighted | 17 | 0.52 (0.30-0.88) | 0.015330016 | 19.817 | 0.229 | NA | NA |
|  | Simple mode | 17 | 0.60 (0.20-1.83) | 0.38234885 | NA | NA | NA | NA |
|  | Weighted mode | 17 | 0.64 (0.25-1.67) | 0.380106426 | NA | NA | NA | NA |
|  | MR-PRESSO | NA | NA | 0.262 | NA | NA | NA | NA |
|  |  |  |  |  |  |  |  |  |
| **BAFF-R on IgD- CD27-** | MR Egger | 14 | 0.76 (0.15-3.75) | 0.741402 | 12.215 | 0.429 | -0.068 | 0.140 |
|  | Weighted median | 14 | 0.61 (0.27-1.38) | 0.235696 | NA | NA | NA | NA |
|  | Inverse variance weighted | 14 | 0.53 (0.29-0.96) | 0.035933 | 12.452 | 0.491 | NA | NA |
|  | Simple mode | 14 | 0.89 (0.25-3.16) | 0.854751 | NA | NA | NA | NA |
|  | Weighted mode | 14 | 0.70 (0.20-2.43) | 0.579926 | NA | NA | NA | NA |
|  | MR-PRESSO | NA | NA | 0.494 | NA | NA | NA | NA |
|  |  |  |  |  |  |  |  |  |
| **CD24 on unsw mem** | MR Egger | 23 | 0.64 (0.34-1.20) | 0.182284 | 16.061 | 0.766 | 0.009 | 0.911 |
|  | Weighted median | 23 | 0.80 (0.44-1.45) | 0.460072 | NA | NA | NA | NA |
|  | Inverse variance weighted | 23 | 0.66 (0.45-0.98) | 0.03726 | 16.073 | 0.812 | NA | NA |
|  | Simple mode | 23 | 0.83 (0.32-2.16) | 0.701208 | NA | NA | NA | NA |
|  | Weighted mode | 23 | 0.81 (0.43-1.53) | 0.516371 | NA | NA | NA | NA |
|  | MR-PRESSO | NA | NA | 0.814 | NA | NA | NA | NA |
|  |  |  |  |  |  |  |  |  |
| **CD3 on CM CD8br** | MR Egger | 16 | 0.63 (0.29-1.43) | 0.296435 | 12.901 | 0.534 | -0.022 | 0.828 |
|  | Weighted median | 16 | 0.64 (0.37-1.11) | 0.113106 | NA | NA | NA | NA |
|  | Inverse variance weighted | 16 | 0.59 (0.40-0.87) | 0.008515 | 12.950 | 0.606 | NA | NA |
|  | Simple mode | 16 | 0.59 (0.25-1.41) | 0.255265 | NA | NA | NA | NA |
|  | Weighted mode | 16 | 0.65 (0.35-1.21) | 0.194637 | NA | NA | NA | NA |
|  | MR-PRESSO | NA | NA | 0.626 | NA | NA | NA | NA |
|  |  |  |  |  |  |  |  |  |
| **CD3 on T cell** | MR Egger | 29 | 0.67 (0.50-0.89) | 0.011050031 | 27.169 | 0.455 | 0.086 | 0.111 |
|  | Weighted median | 29 | 0.91 (0.64-1.29) | 0.587467784 | NA | NA | NA | NA |
|  | Inverse variance weighted | 29 | 0.79 (0.64-0.98) | 0.032724474 | 29.909 | 0.368 | NA | NA |
|  | Simple mode | 29 | 1.47 (0.73-2.99) | 0.293030584 | NA | NA | NA | NA |
|  | Weighted mode | 29 | 0.82 (0.59-1.15) | 0.254556534 | NA | NA | NA | NA |
|  | MR-PRESSO | NA | NA | 0.339 | NA | NA | NA | NA |
|  |  |  |  |  |  |  |  |  |
| **CD127 on CD4+ T** | MR Egger | 8 | 0.50 (0.10-2.42) | 0.419865 | 5.961 | 0.428 | 0.001 | 0.996 |
|  | Weighted median | 8 | 0.52 (0.23-1.19) | 0.121378 | NA | NA | NA | NA |
|  | Inverse variance weighted | 8 | 0.50 (0.27-0.92) | 0.027064 | 5.961 | 0.544 | NA | NA |
|  | Simple mode | 8 | 0.58 (0.19-1.73) | 0.357738 | NA | NA | NA | NA |
|  | Weighted mode | 8 | 0.51 (0.18-1.42) | 0.237972 | NA | NA | NA | NA |
|  | MR-PRESSO | NA | NA | 0.640 | NA | NA | NA | NA |
|  |  |  |  |  |  |  |  |  |
| **CD25 on CD39+ CD4+** | MR Egger | 23 | 1.62 (1.13-2.34) | 0.016886 | 19.362 | 0.562 | -0.127 | 0.142 |
|  | Weighted median | 23 | 1.38 (0.99-1.93) | 0.060645 | NA | NA | NA | NA |
|  | Inverse variance weighted | 23 | 1.31 (1.03-1.65) | 0.026669 | 21.684 | 0.479 | NA | NA |
|  | Simple mode | 23 | 1.08 (0.61-1.93) | 0.798231 | NA | NA | NA | NA |
|  | Weighted mode | 23 | 1.38 (1.02-1.85) | 0.046043 | NA | NA | NA | NA |
|  | MR-PRESSO | NA | NA | 0.528 | NA | NA | NA | NA |
|  |  |  |  |  |  |  |  |  |
| **FSC-A on lymphocyte** | MR Egger | 16 | 0.71 (0.51-0.98) | 0.056791 | 15.810 | 0.325 | -0.020 | 0.762 |
|  | Weighted median | 16 | 0.72 (0.49-1.07) | 0.103615 | NA | NA | NA | NA |
|  | Inverse variance weighted | 16 | 0.60 (0.53-0.90) | 0.006916 | 15.918 | 0.388 | NA | NA |
|  | Simple mode | 16 | 0.85 (0.38-1.87) | 0.687994 | NA | NA | NA | NA |
|  | Weighted mode | 16 | 0.71 (0.53-0.97) | 0.045605 | NA | NA | NA | NA |
|  | MR-PRESSO | NA | NA | 0.523 | NA | NA | NA | NA |
|  |  |  |  |  |  |  |  |  |
| **FSC-A on CD4+ T** | MR Egger | 23 | 0.89 (0.70-1.12) | 0.328053 | 21.870 | 0.407 | -0.097 | 0.132 |
|  | Weighted median | 23 | 0.80 (0.60-1.05) | 0.112283 | NA | NA | NA | NA |
|  | Inverse variance weighted | 23 | 0.80 (0.66-0.98) | 0.032353 | 24.433 | 0.325 | NA | NA |
|  | Simple mode | 23 | 0.81 (0.44-1.50) | 0.508972 | NA | NA | NA | NA |
|  | Weighted mode | 23 | 0.80 (0.63-1.02) | 0.082738 | NA | NA | NA | NA |
|  | MR-PRESSO | NA | NA | 0.351 | NA | NA | NA | NA |
|  |  |  |  |  |  |  |  |  |
| **CD4 on EM CD4+ T** | MR Egger | 19 | 0.50 (0.32-0.80) | 0.00911 | 11.227 | 0.845 | 0.080 | 0.211 |
|  | Weighted median | 19 | 0.61 (0.37-0.99) | 0.043534 | NA | NA | NA | NA |
|  | Inverse variance weighted | 19 | 0.63 (0.47-0.86) | 0.002974 | 12.918 | 0.796 | NA | NA |
|  | Simple mode | 19 | 0.84 (0.43-1.65) | 0.626558 | NA | NA | NA | NA |
|  | Weighted mode | 19 | 0.60 (0.40-0.91) | 0.027731 | NA | NA | NA | NA |
|  | MR-PRESSO | NA | NA | 0.841 | NA | NA | NA | NA |
|  |  |  |  |  |  |  |  |  |
| **CD80 on myeloid DC** | MR Egger | 26 | 0.73 (0.49-1.10) | 0.150798 | 17.396 | 0.831 | 0.012 | 0.834 |
|  | Weighted median | 26 | 0.76 (0.51-1.14) | 0.188632 | NA | NA | NA | NA |
|  | Inverse variance weighted | 26 | 0.76 (0.58-0.99) | 0.048707 | 17.440 | 0.865 | NA | NA |
|  | Simple mode | 26 | 0.86 (0.46-1.67) | 0.663106 | NA | NA | NA | NA |
|  | Weighted mode | 26 | 0.78 (0.52-1.16) | 0.223858 | NA | NA | NA | NA |
|  | MR-PRESSO | NA | NA | 0.897 | NA | NA | NA | NA |
|  |  |  |  |  |  |  |  |  |
| **CD45 on basophil** | MR Egger | 16 | 1.89 (0.82-4.34) | 0.157263 | 7.331 | 0.921 | -0.068 | 0.604 |
|  | Weighted median | 16 | 1.58 (1.01-2.46) | 0.046512 | NA | NA | NA | NA |
|  | Inverse variance weighted | 16 | 1.54 (1.10-2.15) | 0.012805 | 7.612 | 0.938 | NA | NA |
|  | Simple mode | 16 | 1.45 (0.73-2.90) | 0.30589 | NA | NA | NA | NA |
|  | Weighted mode | 16 | 1.56 (0.88-2.76) | 0.147014 | NA | NA | NA | NA |
|  | MR-PRESSO | NA | NA | 0.956 | NA | NA | NA | NA |
|  |  |  |  |  |  |  |  |  |
| **CD8 on NKT** | MR Egger | 17 | 1.08 (0.59-1.97) | 0.81163 | 9.001 | 0.877 | 0.095 | 0.242 |
|  | Weighted median | 17 | 1.30 (0.74-2.26) | 0.354604 | NA | NA | NA | NA |
|  | Inverse variance weighted | 17 | 1.45 (1.01-2.09) | 0.044673 | 10.488 | 0.840 | NA | NA |
|  | Simple mode | 17 | 2.45 (0.97-6.20) | 0.075978 | NA | NA | NA | NA |
|  | Weighted mode | 17 | 1.30 (0.73-2.31) | 0.384486 | NA | NA | NA | NA |
|  | MR-PRESSO | NA | NA | 0.831 | NA | NA | NA | NA |

Note: NA, not applicable. Cochran's Q test (Q-P value >0.05) indicates no heterogeneity exists. The intercept of MR Egger is close to 0 with P >0.05 and MR-PRESSO’s Global test > 0·05 indicates no horizontal pleiotropic outliers.

**Supplementary Table 4. Results of MR and Sensitivity analysis on the association between gut microbiota and immune meditors.**

| **Gut microbiota** | **immune meditors** | **MR method** | **No. of SNP** | **OR (95%CI)** | ***P*-value** | **Heterogeneity test** | | **Pleiotropy test** | |
| --- | --- | --- | --- | --- | --- | --- | --- | --- | --- |
|  |  |  |  |  |  | **Cochran's Q** | ***P*** | **intercept** | ***P*** |
| **Creatinine degradation** | FSC-A on lymphocyte | MR Egger | 3 | 1.69 (0.51-5.59) | 0.54874179 | 2.080 | 0.149 | 0.103 | 0.773 |
|  |  | Weighted median | 3 | 1.28 (0.95-1.71) | 0.103607567 | NA | NA | NA | NA |
|  |  | Inverse variance weighted | 3 | 1.36 (1.07-1.72) | 0.011785359 | 2.368 | 0.306 | NA | NA |
|  |  | Simple mode | 3 | 1.19 (0.85-1.68) | 0.418351248 | NA | NA | NA | NA |
|  |  | Weighted mode | 3 | 1.23 (0.92-1.65) | 0.299785756 | NA | NA | NA | NA |
|  |  | MR-PRESSO | NA | NA | 0.764 | NA | NA | NA | NA |
|  |  |  |  |  |  |  |  |  |  |
| **Creatinine degradation** | FSC-A on CD4+ T | MR Egger | 3 | 1.92 (0.51-7.20) | 0.509288395 | 2.370 | 0.124 | -0.058 | 0.698 |
|  |  | Weighted median | 3 | 1.30 (0.96-1.76) | 0.085856546 | NA | NA | NA | NA |
|  |  | Inverse variance weighted | 3 | 1.38 (1.05-1.81) | 0.022340727 | 2.993 | 0.224 | NA | NA |
|  |  | Simple mode | 3 | 1.20 (0.81-1.77) | 0.463286931 | NA | NA | NA | NA |
|  |  | Weighted mode | 3 | 1.26 (0.91-1.74) | 0.298281637 | NA | NA | NA | NA |
|  |  | MR-PRESSO | NA | NA | 0.615 | NA | NA | NA | NA |
|  |  |  |  |  |  |  |  |  |  |
| **Mannan degradation** | FSC-A on CD4+ T | MR Egger | 6 | 1.10 (0.45-2.73) | 0.841623 | 2.064 | 0.724 | 0.015 | 0.841 |
|  |  | Weighted median | 6 | 1.20 (0.94-1.54) | 0.138508 | NA | NA | NA | NA |
|  |  | Inverse variance weighted | 6 | 1.22 (1.10-1.48) | 0.047857 | 2.110 | 0.834 | NA | NA |
|  |  | Simple mode | 6 | 1.09 (0.78-1.54) | 0.626807 | NA | NA | NA | NA |
|  |  | Weighted mode | 6 | 1.21 (0.90-1.64) | 0.263738 | NA | NA | NA | NA |
|  |  | MR-PRESSO | NA | NA | 0.372 | NA | NA | NA | NA |
|  |  |  |  |  |  |  |  |  |  |
| **L tryptophan biosynthesis** | CD25hi CD45RA+ CD4 not Treg AC | MR Egger | 7 | 1.04 (0.43-2.51) | 0.940291864 | 3.744 | 0.587 | 0.025 | 0.599 |
|  |  | Weighted median | 7 | 1.26 (0.90-1.76) | 0.183463237 | NA | NA | NA | NA |
|  |  | Inverse variance weighted | 7 | 1.32 (1.02-1.70) | 0.032458506 | 4.057 | 0.669 | NA | NA |
|  |  | Simple mode | 7 | 1.27 (0.77-2.10) | 0.382856035 | NA | NA | NA | NA |
|  |  | Weighted mode | 7 | 1.28 (0.80-2.06) | 0.348477325 | NA | NA | NA | NA |
|  |  | MR-PRESSO | NA | NA | 0.789 | NA | NA | NA | NA |
|  |  |  |  |  |  |  |  |  |  |
| **Family_Streptococcaceae** | Tcell %lymphocyte | MR Egger | 8 | 0.57 (0.27-1.21) | 0.195788209 | 6.744 | 0.337 | 0.064 | 0.339 |
|  |  | Weighted median | 8 | 0.82 (0.68-0.98) | 0.037556466 | NA | NA | NA | NA |
|  |  | Inverse variance weighted | 8 | 0.85 (0.74-0.97) | 0.020601665 | 7.955 | 0.345 | NA | NA |
|  |  | Simple mode | 8 | 0.77 (0.58-1.02) | 0.106714978 | NA | NA | NA | NA |
|  |  | Weighted mode | 8 | 0.79 (0.60-1.03) | 0.128945481 | NA | NA | NA | NA |
|  |  | MR-PRESSO | NA | NA | 0.439 | NA | NA | NA | NA |
|  |  |  |  |  |  |  |  |  |  |
| **species_Parabacteroides_goldsteinii** | CD25hi CD45RA+ CD4 not Treg AC | MR Egger | 3 | 0.77 (0.11-5.50) | 0.834943413 | 0.018 | 0.893 | -0.004 | 0.986 |
|  |  | Weighted median | 3 | 0.75 (0.57-0.97) | 0.031012457 | NA | NA | NA | NA |
|  |  | Inverse variance weighted | 3 | 0.75 (0.60-0.93) | 0.009594816 | 0.018 | 0.991 | NA | NA |
|  |  | Simple mode | 3 | 0.74 (0.55-1.02) | 0.205642454 | NA | NA | NA | NA |
|  |  | Weighted mode | 3 | 0.74 (0.55-1.01) | 0.195356369 | NA | NA | NA | NA |
|  |  | MR-PRESSO | NA | NA | 0.456 | NA | NA | NA | NA |
|  |  |  |  |  |  |  |  |  |  |
| **species_Parabacteroides_goldsteinii** | CD127 on CD4+T | MR Egger | 3 | 0.43 (0.05-3.58) | 0.577515098 | 0.058 | 0.810 | 0.097 | 0.712 |
|  |  | Weighted median | 3 | 0.73 (0.54-0.98) | 0.034253015 | NA | NA | NA | NA |
|  |  | Inverse variance weighted | 3 | 0.72 (0.57-0.91) | 0.006371019 | 0.293 | 0.863 | NA | NA |
|  |  | Simple mode | 3 | 0.72 (0.51-1.00) | 0.190262549 | NA | NA | NA | NA |
|  |  | Weighted mode | 3 | 0.74 (0.54-1.01) | 0.200894493 | NA | NA | NA | NA |
|  |  | MR-PRESSO | NA | NA | 0.328 | NA | NA | NA | NA |
|  |  |  |  |  |  |  |  |  |  |
| **species_Alistipes_shahii** | CD25hi %CD4+ Treg | MR Egger | 6 | 1.14 (0.28-4.67) | 0.85724914 | 4.349 | 0.361 | -0.059 | 0.536 |
|  |  | Weighted median | 6 | 0.77 (0.55-1.08) | 0.136120128 | NA | NA | NA | NA |
|  |  | Inverse variance weighted | 6 | 0.71 (0.55-0.92) | 0.010400336 | 4.844 | 0.435 | NA | NA |
|  |  | Simple mode | 6 | 0.80 (0.50-1.27) | 0.387559725 | NA | NA | NA | NA |
|  |  | Weighted mode | 6 | 0.80 (0.54-1.19) | 0.315864135 | NA | NA | NA | NA |
|  |  | MR-PRESSO | NA | NA | 0.065 | NA | NA | NA | NA |
|  |  |  |  |  |  |  |  |  |  |
| **species_Alistipes_shahii** | CD3 on CM CD8+ T | MR Egger | 6 | 1.34 (0.33-5.48) | 0.708705 | 2.062 | 0.724 | 0.089 | 0.413 |
|  |  | Weighted median | 6 | 0.66 (0.45-0.95) | 0.026606 | NA | NA | NA | NA |
|  |  | Inverse variance weighted | 6 | 0.70 (0.53-0.92) | 0.010356 | 2.894 | 0.716 | NA | NA |
|  |  | Simple mode | 6 | 0.59 (0.34-1.04) | 0.127468 | NA | NA | NA | NA |
|  |  | Weighted mode | 6 | 0.59 (0.34-1.02) | 0.11912 | NA | NA | NA | NA |
|  |  | MR-PRESSO | NA | NA | 0.648 | NA | NA | NA | NA |

**Supplementary Figures:**

**Supplementary Figure.1 The scatter plots of MR results between significant gut microbiota/pathway and significant immune meditors.**

**Supplementary Figure.2 The results of medition analysis of metabolic pathway and other microbiota for AOSD. (a).** Mannan degradation exhibited negative regulatory effects on AOSD. **(b).** L-tryptophan biosynthesis exhibited positive regulatory effects on AOSD. **(c-e).** There was no mediation effect existing in the regulations of Creatinine degradation and Species Parabacteroides goldsteinii.
